# Systematics of the *Ceracis furcifer* Species-Group (Coleoptera: Ciidae): The Specialized Consumers of the Blood-Red Bracket Fungus *Pycnoporus sanguineus*

**DOI:** 10.3390/insects8030070

**Published:** 2017-07-17

**Authors:** Italo S. C. Pecci-Maddalena, Cristiano Lopes-Andrade

**Affiliations:** 1 Programa de Pós-Graduação em Ecologia, Departamento de Biologia Geral, Universidade Federal de Viçosa, Viçosa 36570-900, Minas Gerais, Brazil; 2 Laboratório de Sistemática e Biologia de Coleoptera, Departamento de Biologia Animal, Universidade Federal de Viçosa, Viçosa 36570-900, Minas Gerais, Brazil; ciidae@gmail.com

**Keywords:** minute tree-fungus beetles, Neotropical, specialization, host fungus

## Abstract

The *Ceracis furcifer* species-group (Coleoptera: Ciidae) originally comprised nine species names: *Ceracis cornifer* (Mellié, 1849); *C. cylindricus* (Brèthes, 1922); *C. furcifer* Mellié, 1849; *C. hastifer* (Mellié, 1849); *C. monocerus* Lawrence, 1967; *C. ruficornis* Pic, 1916; *C. simplicicornis* (Pic, 1916); *C. semipallidus* Pic, 1922 and *C. unicornis* Gorham, 1898. *Ceracis semipallidus* was synonymised with *C. furcifer* and then no further changes were made to the composition of the group. Here, we provide a taxonomic revision of the *Ceracis furcifer* species-group and new data on the geographic distribution and host fungi of the included species. Lectotypes are designated for *C. cornifer*, *C. furcifer*, *C. hastifer*, *C*. *ruficornis*, *C. semipallidus* and *C. unicornis*. As results we: (i) synonymise *C. cylindricus*, *C. monocerus*, *C. simplicicornis*, *C. unicornis* with *C. cornifer*; (ii) confirm the synonymy of *C. semipallidus* with *C. furcifer*; (iii) redescribe *C. cornifer*, *C. hastifer*, *C. furcifer* and *C. ruficornis*; and (iv) provide an identification key for species in the *furcifer* group. The frontoclypeal horn and body coloration showed great intraspecific variation. We show that species in the *furcifer* group have distributions wider than previously known and use mainly *Pycnoporus sanguineus* as host fungus. Species of the *furcifer* group are the only animals specialized in feeding on basidiomes of *P. sanguineus*.

## 1. Introduction

*Ceracis* was described by Mellié in 1849 [1] as a subgenus of *Ennearthron* Mellié, 1847. Lacordaire [2] elevated *Ceracis* to genus and Lawrence [3] redefined its limits. *Ceracis* was recently redescribed [4] and currently encompasses 56 described species, being the second most speciose genus of Ciidae [4,5,6,7]. Four species-groups were defined for the genus (*C. cucullatus*, *C. furcatus*, *C. furcifer* and *C. singularis* groups) but they encompass only 25 species [3,4,7,8]. Recently, the *C. cucullatus* species-group was partially revised [5,6] and new Australian species were added to the group [4], but systematics of the remaining *Ceracis* species still relies mostly on original descriptions and the revision of North American species [3].

The *Ceracis furcifer* species-group (Coleoptera: Ciidae), as proposed by Lawrence [3], includes nine species names: *Ceracis cornifer* (Mellié, 1849); *C. cylindricus* (Brèthes, 1922); *C. furcifer* Mellié, 1849; *C. hastifer* (Mellié, 1849); *C. monocerus* Lawrence, 1967; *C. ruficornis* Pic, 1916; *C. simplicicornis* (Pic, 1916); *C. unicornis* Gorham, 1898 and *C. semipallidus* Pic, 1922. Hereafter, the *Ceracis furcifer* species-group will simply be called the “*furcifer* group”. When the *furcifer* group was defined, in the same paper Lawrence synonymised *C. semipallidus* with *C. furcifer* [3] and then no changes were made to its composition, leaving the group with eight valid species.

All species of the *furcifer* group have a similar body form, fine and sparse pronotal and elytral punctation, a rounded or shallowly emarginated pronotal apex and a median frontoclypeal horn in males [3]. *Ceracis furcifer* and *C. ruficornis* have eight antennomeres and frontoclypeal horn of the males deeply incised apically, forming two lobes [3], while the other species have nine antennomeres and the frontoclypeal horn of males is rounded, truncated or shallowly emarginate apically. Aside from these features, there are few differences between them, such as colour and dorsal punctation [3]. 

Original descriptions of species of the *furcifer* group provide scant occurrence data, that are sometimes inaccurate and there are few subsequent works citing information on these species. Mellié [1] cited the type-locality of *C. cornifer* solely as “Brésil” (=Brazil) and there are only a few other published records of the species from southeastern Brazil [9]. *Ceracis simplicicornis* was described from Buenos Aires (Argentina) and recorded from São Francisco de Paula (RS-Brazil) [10]. *Ceracis cylindricus*, *C. unicornis*, *C. hastifer* and *C. ruficornis* are known only from the respective type-localities: General Urquiza (in Argentina), St. Vincent (in the Caribbean Sea), Colombia (without further information) and Blumenau (in the state of Santa Catarina, southern Brazil) [1,11,12,13]. The type-locality of *C. furcifer* is Cayenne (in French Guiana) [1] but there are records of this species from Guadeloupe, Surinam, Peru, the Lesser Antilles and “Latin America” [3,14,15,16]. *Ceracis monocerus* was described from Florida (USA) but its distribution extends to Louisiana (USA) and Cuba [3]. It is worth mentioning that the southern tip of Florida is recognised either as part of the Neotropical region or of the Nearctic region [17,18,19,20]. Here, the southern tip of Florida will be treated as part of the Neotropical region, and the surrounding areas as transitions to the Nearctic region. Therefore, all members of the *furcifer* group are essentially Neotropical [3,9,10], with a few records from the Nearctic region [3], and its species have allopatric distributions based on previously known material [3].

There are a few host records of species of the *furcifer* group in *Trametes*, *Coriolus* and *Lenzites* (Basidiomycota: Polyporaceae) [3,21]. However, they have been recorded mostly in the blood-red bracket fungus *Pycnoporus sanguineus* (L.) Murrill (Polyporaceae), a fungus of the *Trametes* ciid host-use group [1,7,9,10,21,22]. *Pycnoporus sanguineus* is widely distributed in the Neotropics [7,10,23,24], being common at open areas in Brazil, as in the Cerrado biome (Brazilian savanna), and forest clearings (pers. obs.), with records also in urban areas [25]. *Pycnoporus sanguineus* is a source of antibiotic compounds for the pharmaceutical industry [26,27,28,29,30] and produces laccase, an enzyme with a wide range of technological applications [31,32,33]. 

A recent molecular phylogenetic analysis shows that *C. cornifer*, *C. furcifer* and *C. simplicicornis* are closely related species but do not cluster with other species of *Ceracis* [34]. Moreover, it shows that *C. cornifer* and *C. simplicicornis* are possibly conspecifics and may well be synonymized [34]. A taxonomic study of the *furcifer* group is necessary to clarify the morphological limits between its species.

Our objective in the present work is two-fold: (i) to provide a taxonomic revision of the *furcifer* group; (ii) to compile data on the use of the fungus *Pycnoporus sanguineus* as a resource by animals and, in this context, discuss the importance of the *furcifer* group as specialized consumers of *P. sanguineus*.

## 2. Material and Methods

At least one syntype each of *C. cornifer*, *C. furcifer*, *C. hastifer* and *C. semipallidus* was examined and dissected. Syntypes of *C. unicornis* were examined but not dissected. The redescription of *C. ruficornis* is based on a plesiotype (specimen used for a redescription, supplementary description, or illustration published after the original description; *sensu* Evenhuis [35]). The type-specimen of *C. cylindricus* was previously considered to be lost [3] and our own attempts to locate it were unsuccessful. We did not examine the type series of either *C. monocerus* or *C. simplicicornis*, but series collected near the type localities of these species were examined. In the case of *C. unicornis*, available images of the syntypes (taken by Dr. Vivian E. Sandoval-Gómez) were compared with examined individuals, and these syntypes were later examined by the senior author. Aside from type series and historical material (see results, material examined), individuals from 64 localities were examined and at least one male from most of these localities was dissected for comparing sclerites of male abdominal terminalia.

Examined beetles belong to the following scientific collections:

**ANIC**Australian National Insect Collection (Canberra, Australia)**BMNH**Natural History Museum (London, UK)**CAMB**Coleção Ayr de Moura Bello (Rio de Janeiro, RJ, Brazil)**CMN**Canadian Museum of Nature (Ottawa, Ontario, Canada)**CNCI**Canadian National Collection of Insects (Ottawa, Ontario, Canada)**CELC**Coleção Entomológica do Laboratório de Sistemática e Biologia de Coleoptera da UFV (Viçosa, MG, Brazil)**CERPE**Coleção Entomológica da Universidade Federal Rural de Pernambuco (Recife, PE, Brazil)**CEMT**Seção de Entomologia da Coleção Zoológica, Departamento de Biologia e Zoologia, Instituto de Biociências, Universidade Federal de Mato Grosso (Cuiabá, MT, Brazil) **DZUP**Coleção Entomologica Pe. J. S. Moure, Universidade Federal do Paraná,(Curitiba, PR, Brazil)**FMNH**Field Museum of Natural History (Chicago, Illinois, USA)**MCNZ**Museu de Ciências Naturais da Fundação Zoobotânica do Rio Grande do Sul (Porto Alegre, RS, Brazil)**MFN**Museum für Naturkunde (Berlin, Germany)**MHNG**Muséum d’Histoire Naturelle (Géneve, Switzerland)**MNHN**Muséum National d’Histoire Naturelle (Paris, France)**MNRJ**Museu Nacional do Rio de Janeiro (Rio de Janeiro, RJ, Brazil)**MPEG**Museu Paraense Emílio Goeldi (Belém, PA, Brazil)**MZSP**Museu de Zoologia da Universidade de São Paulo (São Paulo, SP, Brazil)**NMNH**National Museum of Natural History, Smithsonian Institution (Washington, D. C., USA)**SNSD**Senckenberg Naturhistorische Sammlungen Dresden (Dresden, Germany)

Terms for external morphology and male abdominal terminalia of ciids used here largely follow Lopes-Andrade and Lawrence [36]. We do not include a study of the female abdominal terminalia, due to technical difficulties (e.g., small size and membranous parts) and the absence of morphological features to clearly distinguish females. Therefore, females were not included in the identification key. Range, mean and standard deviation values for measurements (in millimeters) and ratios are provided in redescriptions and the following abbreviations are used: **BW**, basal width of the scutellum; **CL**, length of the antennal club (measured from base of the first antennomere of the club to apex of the last); **EL**, elytral length (at midline, from base of scutellum to elytral apex); **EW**, greatest elytral width; **FL**, length of the antennal funicle (measured from base of the third to apex of the last antennomere before the club); **GD**, greatest depth of the body (from elytra to metaventrite); **GW**, greatest diameter of the eye (measured laterally); **HL**, length of male frontoclypeal horn in lateral view, slightly below baseline of horn (because this is generally concave) to the apex; **PL**, pronotal length along midline; **PW**, greatest pronotal width; and **TL**, total length (=EL + PL; head not included). The ratio **GD/EW** was recorded as an indication of degree of convexity; **TL/EW** indicates degree of body elongation. A maximum of five males and five females from each locality were measured but, in some cases, individuals from close localities were not measured. Differences between specimens are given in the sections on variation, together with standard measurements and ratios. 

Pin label transcriptions are placed within quotation marks, with each label separated by a backslash. Unless otherwise specified (between square brackets), labels are printed on white paper. The number of individuals bearing these labels is stated immediately before label data. The following federal states of Brazil (abbreviations between parentheses) are cited in the text: Amazonas (AM); Bahia (BA); Espírito Santo (ES); Góias (GO); Mato Grosso do Sul (MS); Minas Gerais (MG); Pará (PA); Rio de Janeiro (RJ); Rio Grande do Sul (RS); Santa Catarina (SC); São Paulo (SP); Tocantins (TO). 

Individuals were examined, compared, measured and photographed under a Zeiss Discovery V20 equipped with a Zeiss AxioCam 506 digital camera. Whole mount preparations of male abdominal terminalia followed the protocol described by Lopes-Andrade [37] and photography of dissected pieces was performed under a Zeiss AxioLab compound microscope equipped with a Zeiss AxioCam MRc digital camera. Distribution maps were created in the freeware QGIS 2.12.2 [38], with latitude and longitude coordinates estimated by tracking localities in the online database GeoNames [39]. Maps include all localities of individuals directly examined by us and all previously published records. Maps based on annual temperature means were based on the Bioclim dataset, obtained from a global climate database with high spatial resolution (free climate data for ecological modelling and GIS, version 1.4; www.worldclim.org [40]). 

To compile information on the use of basidiomes of *P. sanguineus* as resource by animals, the terms “*Pycnoporus sanguineus*” and “*Ceracis*” were searched separately in the online databases Web of Knowledge, Scielo, Biodiversity Library and Google Scholar. For each of these databases, we also searched for the terms "*Pycnoporus*" and "*Polyporus sanguineus*" and each of the following terms: “resource”, “consumer”, “consumption”, “Association”, “Interaction”, “Animal” and “Insects”. The term “*Polyporus sanguineus*” was also searched in the database Biodiversity Heritage Library. Additionally, the following combination of terms were searched in Google Scholar and Scielo: (i) “*Ceracis*” and “*Pycnoporus sanguineus*”; (ii) “beetles” and “*Pycnoporus sanguineus*”; (iii) “organisms” and “*Pycnoporus sanguineus*” and “feeding on”; (iv) “organisms” and “*Pycnoporus sanguineus*” and “feed”; (v) “ingestion” and “*Pycnoporus sanguineus*”; (vi) “edible fungus” and “*Pycnoporus sanguineus*”; (vii) “*Boletus sanguineus*” (another synonym of *P. sanguineus*) and “feed”; (viii) “*Polyporus sanguineus*” and “feed”. *Pycnoporus* currently comprises four nearly indistinguishable species [24]. The focus was on *P. sanguineus*, because other *Pycnoporus* species do not occur in the Neotropical region. 

The term “breeding record” considered here follows the definition proposed by previous authors [21,22]. “A ciid species was recorded as breeding in a fungus if at least one of the following criteria was met: the presence in a fungus collection of (1) at least 10 fully pigmented adults; (2) two or more tenerals; or (3) one teneral and two or more mature (fully pigmented) adults” [22]. The term “Tenerals” refers to adults recently eclosed and light-coloured [22]. Breeding records provide robust evidences that the presence of a ciid species in a fungus is not incidental.

## 3. Results

For nomenclatural stability, lectotypes are designated for *C. cornifer*, *C. furcifer*, *C. hastifer*, *C. ruficornis*, *C. semipallidus* and *C. unicornis*. The lectotype of *C. ruficornis* was designated based on annotations made by Dr. John F. Lawrence, a ciid specialist who directly examined the type series in 1965, and photos by Dr. Vivian. E. Sandoval-Gómez, who photographed the lectotype and labels in 2011 and made available the photos to us. Based on patterns of external morphology of adults, including male abdominal terminalia, we propose to **(i)** synonymize *C. cylindricus*, *C. monocerus*, *C. simplicicornis* and *C. unicornis* with *C. cornifer*; **(ii)** confirm the synonym of *C. semipallidus* with *C. furcifer* previously proposed by Lawrence [3]; **(iii)** provide redescriptions for *C. cornifer*, *C. hastifer*, *C. furcifer* and *C. ruficornis*, here considered valid species of the *furcifer* group; **(iv)** provide an identification key for *furcifer* group species. Evidence and arguments for the taxonomic acts proposed here are provided in the Species Accounts, especially with respect to the new synonyms of *C. cornifer* (see “Remarks”).

The records of animals feeding on basidiomes of *P. sanguineus* are compiled in Table A1, together with breeding records when available. Table A2 provides information on all other host fungi used by *furcifer* group.

### 3.1. Taxonomic synopsis

#### 3.1.1. *Ceracis cornifer* (Mellié, 1849)

*Ennearthron cylindricum* Brèthes, 1922, **new synonym**

*Ceracis monocerus* Lawrence, 1967, **new synonym**

*Ennearthron simplicicorne* Pic, 1916, **new synonym**

*Ceracis unicornis* Gorham, 1898, **new synonym**

#### 3.1.2. *Ceracis furcifer* Mellié, 1849

Ceracis semipallidus Pic, 1922

#### 3.1.3. *Ceracis hastifer* (Mellié, 1849)

#### 3.1.4. *Ceracis ruficornis* Pic, 1916

**Diagnosis of the *furcifer* group**. The frontoclypeal ridge in males is usually strongly produced forming a median horn, generally laminar in lateral view and bearing several minute, sparse setae; the horn is of variable length, apically bifurcated or not, almost absent in small males to longer than pronotal length in the largest males (Figure 5). The antennal funicle has three or four antennomeres, leading to a total of eight or nine antennomeres in each antenna but never 10. The prosternal process is thin, parallel-sided, but not laminate, as in other *Ceracis*. In male abdominal terminalia, the posterior edge of sternite VIII has a deep, mesal concave emargination; the tegmen has a deep, apical longitudinal emargination forming two wide parallel lobes with rounded or blunt apices; the penis is cylindrical with rounded apex.

**Remarks**. Although species in the *furcifer* group possess most of the diagnostic characteristics currently accepted for the genus *Ceracis*, some remarkable aspects distinguish this group within the genus and may be used to define a separate genus in the future: 

**(i)** Frontoclypeal ridge strongly produced forming a single median horn (bifurcate or not at apex), laminar in lateral view and bearing several minute sparse setae along it. The “false *furcifer* species”, *C. zarathustrai* Pecci-Maddalena et al., 2014 also has a single frontoclypeal horn but it is subcylindrical and bears a conspicuous tuft of yellowish bristles at apex of the frontoclypeal horn [7]. Species of the *Ceracis cucullatus* group also have the frontoclypeal ridge produced forward, forming a short and wide lamina [6] but not a horn. In species of other ciid genera, such as *Grossicis* Antunes-Carvalho et al., males have a laminate frontoclypeal projection, which is wide and conspicuously produced upwardly in *Grossicis diadematus* (Mellié, 1849) but is relatively narrow in *G. laminicornis* Antunes-Carvalho et al. However, in the latter species the horn is curved in lateral view [41] and not as narrow as in *furcifer* group; **(ii)** terminal sclerites of the male abdominal terminalia are similar in *furcifer* group species but the tegmen is different from that of other *Ceracis*. In the *furcifer* group, the tegmen has a deep, apical longitudinal emargination forming two parallel lobes, with rounded or blunt apices; the penis is cylindrical with rounded apex; and sternite VIII has a conspicuous, deep concave, mesal emargination at the posterior edge. In *C. zarathustrai* and in species of the *cucullatus* group, sternite VIII has a shallow V-shaped emargination at the posterior edge, the lateral lobes of tegmen are narrow and acute at apex and the penis has a triangular sclerotisation at the middle of the apical portion [6,7]; **(iii)** prosternal process thin but not laminate (Figure 4A and 11.2, white arrows). Most species of *Ceracis* can be easily distinguished from species of *Cis* and most other Ciinae by the possession of spinose prothoracic tibial apices and a concave prosternum with laminate prosternal process [3]. In species of *Ceracis*, the prosternal process is laminate (Figure 11.1, white arrows), except for species in the *furcifer* group and in *C. bifurcus* Gorham, 1898, *C. laticornis* Pic, 1922, *C. particularis* Pic, 1922 and *C*. *taurulus* Jaquelin du Val, 1857, in which the prosternal process is thin and parallel-sided. 

**Figure 1:**
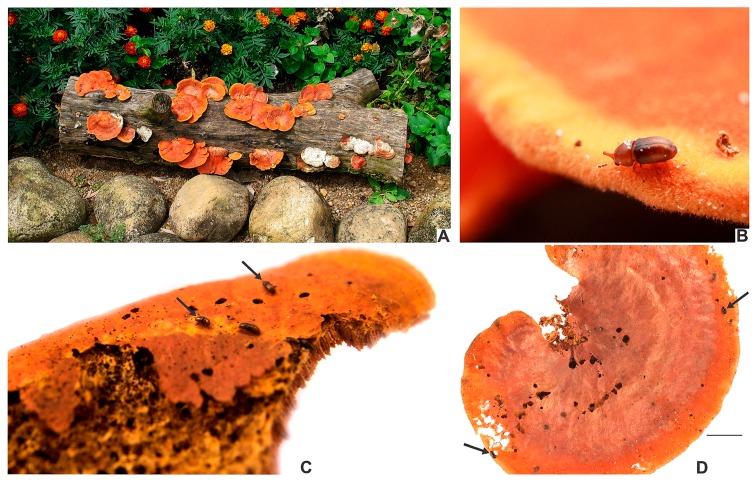
***Pycnoporus sanguineus* (L.) Murrill, host fungus of species of the *furcifer* group.** (**A**) Basidiomes at a garden in Juiz de Fora, Minas Gerais (MG), Brazil. (**B**) A male of *C. cornifer* (Mellié) species on a *P. sanguineus* basidiome from Serra do Cipó, MG, Brazil. **C−D** Basidiomes colonized (**C**) by individuals of *Ceracis cornifer* (Mellié) from Juiz de Fora (arrows indicating the presence of two) and (**D**) by individuals of *Ceracis furcifer* Mellié from Araguaína, Tocantins (TO), Brazil (arrows). Figure 1D, scale bar = 0.5 mm.

**Ecology**. Species of the *furcifer* group are frequently found in basidiomes of *P*. *sanguineus* (Figure 1, Table A1), with incidental records in other fungi (Table A2). 

**Figure 2:**
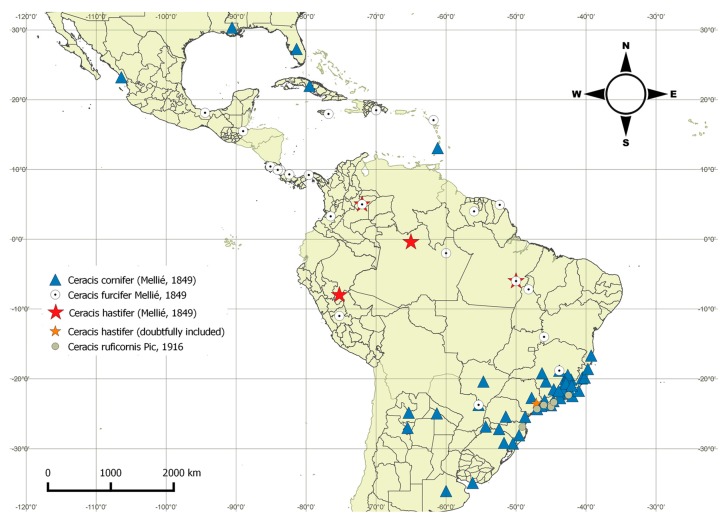
**Distribution map for species of the *furcifer* group.** The doubtful record of *Ceracis hastifer* (orange star) is based on individuals from São Roque (São Paulo (SP), Brazil). Note: the distribution of *C. cornifer* is disjunct. Overlapping symbols represent localities where two species are sympatric: *C. cornifer* and *C. furcifer* in Paranhos (Mato Grosso do Sul (MS), Brazil) and Serra do Cipó (MG, Brazil); *C. furcifer* and *C. hastifer* in San Luis de Palenque (Casanare, Colombia) and Marabá (Pará (PA), Brazil); and *C. ruficornis* and *C. cornifer* in Paraty (Rio de Janeiro (RJ)), Ilha da Vitória (SP) and Peruíbe (SP), Southeast Brazil.

**Distribution.** Neotropical region, from southern Argentina to southern USA (Figure 2). 

### 3.2. Species Accounts

#### 3.2.1. *Ceracis cornifer* (Mellié, 1849)

**Figure 3:**
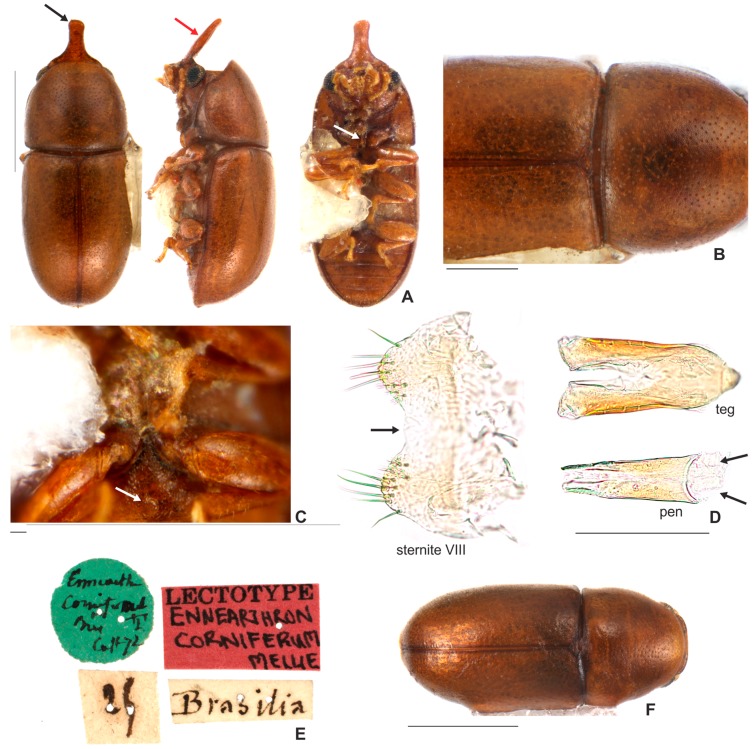
***Ceracis cornifer* (Mellié). A−E** Male lectotype, (**A**) dorsal, lateral and ventral views, respectively. Frontoclypeal horn subtruncate at apex (black arrow) and laminate in lateral view (red arrow); prosternal process thin but not laminate (white arrow), (**B**) pronotal punctation, (**C**) first abdominal ventrite with a sex patch at the centre (white arrow), (**D**) male terminalia, showing sternite VIII (with a deep concave emargination at middle, black arrow), tegmen (teg), penis (pen, basal edge blunts; black arrows), (**E**) labels of the lectotype deposited in the Muséum National d’Histoire Naturelle, MNHN (Paris, France), (**F**) female plesiotype from Viçosa (MG, Brazil). Scale bars: A = 0.5 mm, B = 0.2 mm, C,D = 0.1 mm, F = 0.5 mm.

**Figure 4:**
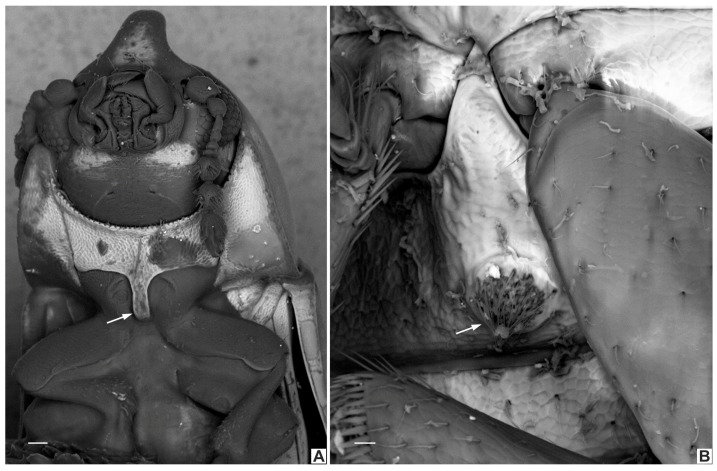
*Ceracis cornifer* (Mellié, 1849), SEM under variable pressure and without any sputter-coating of male specimen from Ubá (MG, Brazil). (**A**) Prosternal process thin but not laminate (white arrow), (**B**) first abdominal ventrite with a sex patch (white arrow). Scale bars: A = 0.03 mm, B = 0.01 mm.

**Figure 5:**
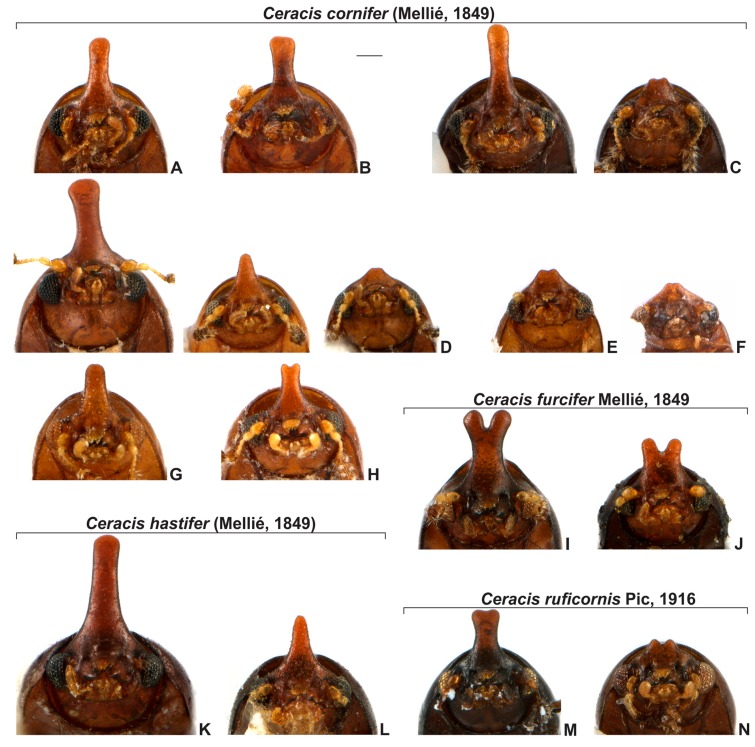
**Male morphs of species of the *furcifer* group from different localities. A−H** *Ceracis cornifer* (Mellié), (**A**) lectotype from Brazil, (**B**) Florida, United States of America, (**C**) Urubici, SC, Brazil, (**D**) Juiz de Fora, MG, Brazil, (**E,F**) Jequeri, MG, Brazil, (**G**) São Francisco de Paula, RS, Brazil, (**H**) Tucuman, Argentina. **I,J** *Ceracis furcifer* Mellié, (**I**) Paranhos, MS, Brazil, (**J**) Colombia. **K,L**, *Ceracis hastifer* (Mellié), (**K**) lectotype from Colombia, (**L**) Colombia. **M,N** *Ceracis ruficornis* Pic, (**M**) Peruíbe, SP, Brazil, (**N**) Ilha dos Búzios, SP, Brazil. Scale bar: 0.1 mm.

**Figure 6:**
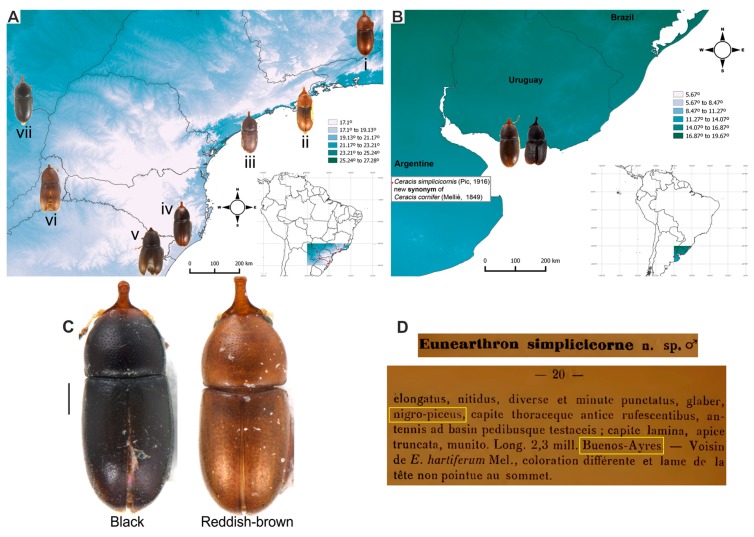
**Colour variation in individuals of *Ceracis cornifer* (Mellié).** (**A**) Localities in a temperature map (in Celsius degrees): (i) Viçosa, MG (Brazil), (ii) Ilha da Vitória, SP (Brazil), (iii) Peruibe, SP (Brazil), (iv) Urubici, SC (Brazil), (v) São Francisco de Paula, RS (Brazil), (vi) Wanda, Misiones (Argentina), (vii) Paranhos, MS, Brazil. (**B**) Specimens from the southern tip of the distribution. (**C**) Coloration black and reddish-brown. (**D**) Original description of *C. simplicicornis* Pic (new synonym of *C. cornifer* proposed here) from Buenos Ayres as “nigro-piceus”.

Figure 1B,C; Figure 3A–F; Figure 4; Figure 5A–H; Figure 6A–C and Figure 11.5.

*Ennearthron corniferum* Mellié, 1849: 371, pl. 4, Figure 18 [1]. Type locality: Brésil; Blackwelder 1945: 549 [14] {distribution}; Casey 1898: 90 [42] {taxonomic notes}; Lawrence 1967: 97 [3] {taxonomic status}; Abdullah 1973: 198 [15] {taxonomic status and distribution}.

*Ceracis cornifer* (Mellié, 1849): Lawrence 1967: 99 [3] {taxonomic notes}; Abdullah 1973: 198 [15] {taxonomic status and distribution}; Lopes-Andrade 2002: 6 [43] {taxonomic notes}; Gumier-Costa et al., 2003: 359 [9] {host fungus and distribution}; Graf-Peters et al. 2011: 558 [10] {host fungi and feeding habit}; Antunes-Carvalho and Lopes-Andrade 2011: 61 [5] {taxonomic notes}; Pecci-Maddalena et al. 2014: 482 [7] {taxonomic notes}; Lopes-Andrade and Grebennikov 2015: 476 [34] {molecular data}

*Ceracis unicornis* Gorham, 1898: 332, **syn. n.** Type locality: Saint Vincent, West Indies [12]; Blackwelder 1945: 550 [14] {distribution}. Lawrence 1967: 98 [3] {taxonomic status}; Antunes-Carvalho and Lopes-Andrade 2011: 61 [5] {taxonomic notes}; Abdullah 1973: 203 [15] {taxonomic status and distribution}; Peck 2015: 149 {distribution} [16].

*Ennearthron simplicicorne* Pic, 1916: 19 **syn. n.** Type locality: Buenos Aires, Argentina [13]; Blackwelder 1945: 550 [14] {distribution}; Lawrence 1967: 97 [3] {taxonomic status}; Abdullah 1973: 202 [15] {taxonomic status and distribution}.

*Ceracis simplicicornis* (Pic, 1916): Lawrence 1967: 99 [3] {taxonomic notes}; Graf-Peters et al. 2011: 556 [10] {feeding habitat}; Antunes-Carvalho and Lopes-Andrade 2011: 61 [5] {taxonomic notes}; Pecci-Maddalena et al. 2014: 482 [7] {taxonomic notes}; Abdullah 1973: 202 [15] {taxonomic status and distribution}; Lopes-Andrade and Grebennikov 2015: 476 [34] {molecular data}

*Ennearthron cylindricum* Brèthes, 1922: 303, **syn. n.** Type locality: General Urquiza, Argentina [11]; Blackwelder 1945: 549 [14] {distribution}; Lawrence 1967: 97 [3] {taxonomic status; location of type unknown}; Abdullah 1973: 199 [15] {taxonomic status and distribution}.

*Ceracis cylindricus* (Brèthes, 1922): Lawrence 1967: 99 [3] {taxonomic notes}; Antunes-Carvalho and Lopes-Andrade 2011: 61 [5] {taxonomic notes}; Pecci-Maddalena 2014: 482 [7] {taxonomic notes}; Abdullah 1973: 199 [15] {taxonomic status and distribution}. 

*Ennearthron unicorne* Casey, 1898: 90 [42]; *Ceracis monocerus*, new name, Lawrence, 1967: 115, Figure 20, **syn. n.** Type locality: Lake placid, Highlands, Florida, United States [3]; Lawrence 1973: 202 [21] {feeding habitat}; Gumier-Costa et al., 2003: 359 [9] {host fungus and distribution}; Graf-Peters et al. 2011: 563 [10] {feeding habit}; Antunes-Carvalho and Lopes-Andrade 2011: 61 [5] {taxonomic notes}; Pecci-Maddalena et al. 2014: 486 [7] {taxonomic notes}; Abdullah 1973: 200 [15] {taxonomic status and distribution}.

**Diagnosis.** Each lateral contour of male frontoclypeal horn usually bears a pronounced inflection near base; the horn is elongate, with rounded to subtruncate apex. The antennal funicle has four antennomeres in which the first is shorter than the next three together. The pronotum is convex with a broadly rounded anterior edge. The anterior portion of each hypomeron has a slightly rounded outer edge. The tegmen has a narrow inner emargination at the apical edge which is less than one half of the total length of the tegmen; its outer edges bear an inflection at the first basal one half; each lobe of the apical portion has one small denticle at the inner apical edge and a slight notch on the outer portion.

**Redescription. Lectotype, here designated (Figure 3A–E).** Adult male apparently fully pigmented. Measurements (in mm): TL 1.21, PL 0.43, PW 0.53, EL 0.78, EW 0.56, GD 0.47. Ratios: PL/PW 0.80, EL/EW 1.38, EL/PL 1.82, GD/EW 0.84, TL/EW 2.14. **Body** glabrous, elongate, subcylindrical; dorsum mostly yellowish-brown; venter mostly reddish-brown; basal antennomeres and mouthparts yellowish brown; antennal funicle, tarsi and legs yellowish to reddish-brown; antennal club dark reddish-brown. **Head** barely visible from above; area immediately above horn base concave, glabrous, sparsely punctate; frontoclypeal ridge strongly produced forming a long, narrow median horn directed upwardly (in mm: length 0.29; basal width 0.28, Figure 3A, black arrow), laminate in lateral view (Figure 3A, red arrow) and bearing several minute, sparsely distributed setae; horn with a pronounced lateral inflection near base (Figure 11.5, big black arrow) and a rounded, extended subtruncate apex (Figure 3A and 11.5). **Antennae** (left antenna measured) with FL 0.08 mm, CL 0.15 mm, CL/FL1.9, length of antennomeres I−IX (in mm) as follows: 0.06, 0.04, 0.03, 0.02, 0.01, 0.01, 0.05, 0.05, 0.05; sensillifers barely visible. **Eyes** coarsely faceted, with minute slender yellowish setae in intersections of ommatidia; GW 0.12 mm. **Pronotum** with anterior edge uniformly rounded and covering the head; posterior edge sublinear, with fine, single punctation; punctures uniform and regularly distributed (Figure 3B); distance between punctures about 4 to 5 puncture-widths; each puncture bearing a yellowish decumbent minute setae, barely visible even at a magnification of 150x; interspaces microreticulate. **Scutellum** small, subtriangular; BW 0.08 mm and SL 0.04 mm. **Elytra** about 1.8x as long as pronotum; sides subparallel at basal two-thirds, then abruptly converging toward apex; punctation single, similar to pronotal punctation but comparatively finer; vestiture similar to that of pronotum; humeral calli conspicuous. **Metathoracic wings** developed, apparently functional. **Prosternum** in front of coxae shallowly biconcave; prosternal process thin (Figure 3A lectotype and Figure 4A other specimen white arrow), parallel-sided, as long as coxae and projected below prosternal disc. **Hypomera** subglabrous and microreticulated, biconcave, anterior portion with outer edge slightly rounded (Figure 11.5, small black arrow). **Pro-, meso- and metathoracic tibiae** as in the diagnosis of the *furcifer* group. **Metaventrite** microreticulate, moderately convex, subglabrous, bearing scattered slender setae; discrimen not discernible in this specimen. **Abdominal ventrites** microreticulate; punctation shallow; vestiture of scattered slender setae, longer than those on dorsum; length of ventrites I−V (in mm, from base to apex of each ventrite at the longitudinal midline): 0.16, 0.06, 0.07, 0.07, 0.07. First abdominal ventrite bearing a circular, marginated, pubescent sex patch at centre (Figure 3C lectotype and 4B other specimen, white arrow) with a transverse diameter of 0.03 mm in the lectotype specimen. **Male abdominal terminalia** (Figure 3D, lectotype) with posterior edge of **sternite VIII** bearing a deep, concave, mesal emargination (Figure 3D, sternite VIII, black arrow); posterior corners sclerotised and rounded, bearing bristles; disc membranous; lateral edges diverging from posterior to anterior portion; anterior edge sublinear. **Tegmen** (Figure 3D, teg and Figure 11.5, teg) with inner emargination of apical edge narrow and shallow, less than half total length (Figure 11.5, teg, red dashed line); each lobe of the apical portion bearing one small denticle at the inner apical edge (Figure 11.5, teg, black arrow) and a small notch on the outer portion (Figure 11.5, teg, blue arrow); lateral edges with an inflection at the basal half (Figure 11.5, teg, red arrow). **Penis** elongate, subcylindrical, basal edge blunt (Figure 3D, pen, black arrows), sides subparallel along basal two-thirds and then gradually converging apically.

**Females** (Figure 3F). Similar to males except for the following features: abdominal sex patch absent; anterior edge of head shallowly emarginated; frontoclypeal ridge devoid of horn.

**Variation.** Males, measurements in mm (n = 79, included the lectotype) TL 0.95−1.41 (1.17 ± 0.09); PL 0.34−0.52 (0.42 ± 0.03); PW 0.4−0.6 (0.5 ± 0.04); EL 0.61−0.94 (0.74 ± 0.07); EW 0.44−0.66 (0.53 ± 0.04); GD 0.38−0.55 (0.46−0.04); HL 0.01−0.41 (0.25 ± 0.11). Ratios: PL/PW 0.75−0.93 (0.85 ± 0.04); EL/EW 1.25−1.59 (1.4 ± 0.07); EL/PL 1.47−2.14 (1.74 ± 0.14); GD/EW 0.74−0.94 (0.86 ± 0.03); TL/EW 2.08−2.41 (2.2 ± 0.07). Females, measurements in mm (n = 65) TL 0.95−1.47 (1.19 ± 0.1); PL 0.35−0.55 (0.43 ± 0.04); PW 0.4−0.6 (0.48 ± 0.04); EL 0.6−0.96 (0.76 ± 0.07); EW 0.44−0.66 (0.53 ± 0.04); GD 0.38−0.67 (0.46−0.05); Ratios: PL/PW 0.8−1 (0.89 ± 0.04); EL/EW 1.25−1.54 (1.43 ± 0.07); EL/PL 1.4−2.2 (1.78 ± 0.16); GD/EW 0.78−0.94 (0.87 ± 0.03); TL/EW 2.02−2.38 (2.23 ± 0.06). Individuals of the following localities were measured (localities between parentheses): **BRAZIL:** State of Minas Gerais (Carrancas, Guaraciaba, Ipatinga, Jequeri, Juiz de Fora, Pains, Piau, Rio Paranaíba, Sacramento, Ubá, Viçosa); State of Rio de Janeiro (Grussaí and Seropédica); State of Espírito Santo (Alto Bergamo and Conceição da Barra); State of São Paulo (Ilha da Victoria); State of Mato Grosso do Sul (Campo Grande and Paranhos); State of Santa Catarina (Urubici). **URUGUAY:** Montevideo. **ARGENTINA:** Tucuman. **MEXICO:** Mazatlán. **UNITED STATES:** Florida (Lake Placid Highlands).

**Material examined. Lectotype male, here designated. (MNHN; Figure 3A)** “*Ennearthron corniferum*? Cast 72? [circular green label; handwritten]\25? [handwritten]\Brasilia [handwritten]\LECTOTYPE *Ennearthron corniferum* [red label; handwritten]”. **Historical Material:** *Ceracis unicornis*, lectotype male, here designated. (**BMNH**) “Type [red. marg. disc; printed]\type [red label; handwritten]\St, Vincent, W.I., H.H., Smith [printed], 247. [handwritten]\W. Indies. 98.237. [printed]\Ceracis unicornis Gorh. ♂. [handwritten]”; 1 paralectotype female, here designated. (**BMNH**) “W. Indies. 98.237. [printed]\St, Vincent, W.I., H.H., Smith [printed], 247. [handwritten]\Paratype? [yellow. marg. disc; printed]\Ceracis unicornis Gorh. ♀. [handwritten]”; 1 specimen male (**BMNH**) “St, Vincent, H.H., Smith, 99-37. [printed]; 1 specimen female (**BMNH**) “St, Vincent, H.H., Smith, 99-37. [printed]\2 [pencilled note-details of collecting data]”. **BRAZIL.** 3 specimens (undetermined locality) (2 ANIC; 1 CELC) “BRAZIL J. Rick\J.F. Lawrence Lot. 1986\Polyporus haedinus\ex. USDA. Herbaria”. **State of Minas Gerais:** 13 specimens (CELC) “Brazil: MG, PN Serra do Cipó, próx. à Cachoeira da Farofa, -19.385724, -43. 585808, 11.v.2016, 758 m, Em grande tronco podre caído, leg. J. Chaul & E. Epifânio\ex *Pycnoporus sanguineus*; 51 specimens (2 CNCI; 2 CERPE; 47 CELC, including one dissected male) “Brazil: MG, Juiz de Fora, Embrapa, 20.ix.2013, Pecci-Maddalena, Í.S.C. leg.\ex. *Pycnoporus sanguineus*”; 16 specimens (CELC, including 1 dissected male) “Brazil: MG, Jequeri, Grota, 20.i.2011, Sandoval-Gómez, V.E. leg.\ex. *Pycnoporus sanguineus*”; 15 specimens (CELC, including one dissected male) “Brazil: MG, Jequeri, Piscamba, vi.2010, Edigio, E.M. leg.\ex. *Pycnoporus sanguineus*”; 33 specimens (2 CMN; CELC, including 1 dissected male) “Brazil: MG, Sacramento, Distrito de Manhuaçu, 15.vii.2010, Antunes-Carvalho, C. leg.\ex. *Pycnoporus sanguineus*”; 32 specimens (CELC, including one dissected male) “Brazil: MG, Sacramento, Distrito de Manhuaçu, 18.vii.2010\Antunes-Carvalho, C. leg.\ex. *Pycnoporus sanguineus*”; 30 specimens (2 FMNH; CELC, including one dissected male) “Brazil: MG, Sacramento, Distrito de Manhuaçu, 31.vii.2010\Antunes-Carvalho, C. leg.\ex. *Pycnoporus sanguineus*”; 32 specimens (CELC, including one dissected male) “Brazil: MG, Viçosa, UFV-Apiário, 09.vi.2010, Campos, L.A. leg.\ex. *Pycnoporus sanguineus*”; 13 specimens (CELC, including 1 dissected male) “Brazil: MG, Viçosa, Campus UFV, 02.i.2008, Campos, L.A. leg.\ex. *Pycnoporus sanguineus*”; 1 specimen (CELC) “Brazil: MG, Viçosa, Violeira, 17.xii.2004, Zacaro, A.A. leg.”; 5 specimens (1 CAMB; 4 CELC) “Brazil: MG, Viçosa, Atrás do insetário, 14.xi.2003, Zacaro, A.A. & Lopes-Andrade, C. legs. [sic]”; 38 specimens (2 ANIC; 36 CELC, including one dissected male) “Brazil: MG, Pains, 01.vi.2008, Soares, L.G.S. leg.\ex. *Pycnoporus sanguineus*”; 30 specimens (2 ANIC; 28 CELC) “Brazil: MG, Rio Paranaiba, Cerrado, ES Ponto 11, CE 11 Tronco caído 24 cm Pote2, 05.i.2011, Resende, N.F. leg.\ex. *Pycnoporus sanguineus*”; 30 specimens (2 ANIC; 28 CELC) “Brazil: MG, Rio Paranaiba, Cerrado, ES Ponto 11, CE 11 Tronco caído 18 cm Tempo sol, 05.i.2011\Resende, N.F. leg.\ex. *Pycnoporus sanguineus*”; 16 specimens (CELC, including one dissected male) “Brazil: MG, Rio Paranaiba, Cerradão C2 (19 cm) Fotos 45-46 6.27 g, 26.xii.2011\Resende, N.F. leg.\ex. *Pycnoporus sanguineus*”; 1 specimen (CELC) “Brazil: MG, Rio Paranaiba, Cerradão C1 (27 cm) Fotos 47-48-49 107.79 g, 26.xii.2011\Resende, N.F. leg.\ex. *Pycnoporus sanguineus*”; 8 specimens (CELC) “Brazil: MG, Rio Paranaiba, Cerradão C6 (11 cm) Fotos 291-292 2.32 g, 28.xii.2011, Resende, N.F. leg.\ex. *Pycnoporus sanguineus*”; 7 specimens (CELC, including one dissected male) “Brazil: MG, Rio Paranaiba, Cerradão C7 (18 cm) Fotos 52-53 13.54 g, 28.xii.2011\Resende, N.F. leg.\ex. *Pycnoporus sanguineus*”; 3 specimens (CELC, including one dissected male) “Brazil: MG, Rio Paranaiba, Campo limpo P7 (20 cm) Fotos 25-26-27 56.22 g, 14.xii.2011\Resende, N.F. leg.\ex. *Pycnoporus sanguineus*”; 2 specimens (CELC, including one dissected male) “Brazil: MG, Rio Paranaiba, Campo limpo P1 (30 cm) Fotos 5-6 4.82 g, 12.xii.2011, Resende, N.F. leg.\ex. *Pycnoporus sanguineus*”; 2 specimens (CELC) “Brazil: MG, Rio Paranaiba, Campo limpo P2 (14 cm), Fotos 8-9 12,18 cm, 12.xii.2011, Resende, N.F. leg.\ex. *Pycnoporus sanguineus*”; 2 specimens (CELC) “Brazil: MG, Rio Paranaiba, Campo limpo P1 (15 cm) Fotos 1-2-3-4 5.31 g, 12.xii.2011\Resende, N.F. leg.\ex. *Pycnoporus sanguineus*”; 10 specimens (CELC) “Brazil: MG, Rio Paranaiba, Cerrado ES Ponto 11 CE 11 tronco caído 24 cm tempo sol, 05.i.2011\Resende, N.F. leg.\ex. *Pycnoporus sanguineus*”; 10 specimens (CELC) “Brazil: MG, Rio Paranaiba, Ponto 11 CE 11 tronco caído 18 cm tempo sol, 05.i.2011\Resende, N.F. leg.\ex. *Pycnoporus sanguineus*”; 6 specimens (CELC, including one dissected male) “Brazil: MG, Rio Paranaiba, Cerrado ES Ponto 10 CE 10 tronco caído 18 cm tempo sol, 05.i.2012, Resende, N.F. leg.\ex. *Pycnoporus sanguineus*”; 15 specimens (CELC) “Brazil: MG, Rio Paranaiba, Cerrado ES 10 CE 11 Fotos 57-58 18 cm 9.039 g, 05.i.2012, Resende, N.F. leg.\ex. *Pycnoporus sanguineus*”; 6 specimens (CELC) “Brazil: MG, Rio Paranaiba, Cerrado ES CE11 Fotos 1701-1702-1703 28 cm 1.08 g, 05.i.2012, Resende, N.F. leg.\ex. *Pycnoporus sanguineus*”; 14 specimens (CELC) “Brazil: MG, Rio Paranaiba, Cerrado ES CE11 Fotos 59-60 24 cm 45.57 g, 05.i.2012, Resende, N.F. leg.\ex. *Pycnoporus sanguineus*”; 11 specimens (CELC, including one dissected male) “Brazil: MG, Rio Paranaiba, Cerrado ES CE8 (28 cm) Fotos 310-311 50.74 g, 03.i.2012, Resende, N.F. leg.\ex. *Pycnoporus sanguineus*”; 1 specimen (CELC) “Brazil: MG, Rio Paranaiba, Cerrado ES CE11 Fotos 217-218-219 16 cm 50.38 g, 05.i.2012, Resende, N.F. leg.\ex. *Pycnoporus sanguineus*”; 2 specimens (CELC) “Brazil: MG, Rio Paranaiba, Cerrado ES CE10 Fotos 315-316 18 cm 129.014 g, 05.i.2012\Resende, N.F. leg.\ex. *Pycnoporus sanguineus*”; 40 specimens (CELC) “Brazil: MG, Rio Paranaiba, Cerrado ES árvore vinho/tronco caído 20 cm Ponto 12 CE 12, 05.i.2012\Resende, N.F. leg.\ex. *Pycnoporus sanguineus*”; 25 specimens (CELC) “Brazil: MG, Rio Paranaiba, Cerrado ES Ponto 10 CE 10 Tronco caído 18cm tempo sol, 05.i.2012, Resende, N.F. leg.\ex. *Pycnoporus sanguineus*”; 4 specimens (CELC) “Brazil: MG, Rio Paranaiba, Cerrado ES Ponto 10 Tronco caído 18cm, 05.i.2012, Resende, N.F. leg.\ex. *Pycnoporus sanguineus*”; 10 specimens (CELC) “Brazil: MG, Rio Paranaiba, Cerrado ES Ponto 10 (CE 10) tronco caído 18 cm, tempo sol, 05.i.2012, Resende, N.F. leg.\ex. *Pycnoporus sanguineus*”; 39 specimens (6 ANIC; 33 CELC, including one dissected male) “Brazil: MG, Ipatinga, 2006, Nolasco, J.P. leg.\ex. *Pycnoporus sanguineus*”; 33 specimens (CELC, including one dissected male) “Brazil: MG, Ipatinga, 2009, Nolasco, J.P. leg.\ex *Pycnoporus sanguineus*”; 2 specimens (CELC, including one dissected male) “Brazil: MG, Ipatinga, RPPN ZACA, 07.xii.2011, Araújo, L.S. leg.\código: Trans: 2 Parcela: C Fungo 1”; 18 specimens (CELC, including one dissected male) “Brazil: MG, Ipatinga, 2009, Nolasco, J.P. leg.\ex. *Pycnoporus sanguineus*”; 21 specimens (2 ANIC; 2 CAMB; 17 CELC, including one dissected male) “Brazil: MG, Ubá, Faz. Córrego do Pari (S21°08’’W42°32’, 311m), viii.2000, Gumier-Costa, F. leg.\*Ceracis cornifer* (Mellié, 1848) det. Cristiano Lopes-Andrade 2003”; 2 specimens (CELC) “Brazil: MG, Ubá, Faz. Córrego do Pari, x.2000, Gumier-Costa, F. leg.\*Ceracis cornifer* (Mellié, 1848) det. Cristiano Lopes-Andrade 2003”; 2 specimens (CELC) “Brazil: MG, Ubá, Faz. Córrego do Pari, x.2000, Gumier-Costa, F. leg.\*Ceracis cornifer* (Mellié, 1848) det. Cristiano Lopes-Andrade 2003\Fotografado em MEV-PV; Piracicaba SP, NAP/MEPA (ESALQ/USP), VII / 2002; C. Lopes-Andrade”; 22 specimens (CELC, including one dissected male) “Brasil: MG, Piau, 22.vi.2014, Pecci-Maddalena, Í.S.C. leg.\ex. *Trametes* sp.”; 7 specimens (CELC) “Brasil: MG, Piau, 22.vi.2014, Pecci-Maddalena, Í.S.C. leg.\ex. *Pycnoporus sanguineus*”; 13 specimens (CELC, including one dissected male) “Brasil: MG, Carrancas, Complexo da Zilda, cerrado e fragmento de mata 27-29.xii.2012, Oliveira, E.H. leg.”; 12 specimens (CELC, including one dissected male) “Brasil: MG, Guaraciaba, Chalé do Turvo, 21.v.2012, Lopes-Andrade, C. leg.\ex. *Pycnoporus sanguineus*”; 20 specimens (CELC) “Brasil: MG, Texeiras, “saindo de Viçosa no restaurante da BR, 12.i.2014, Lopes-Andrade, C. leg.\ex. *Pycnoporus sanguineus*”; 10 specimens (CELC) “Brasil: MG, Ibitipoca, 03.iii.2014, Pecci-Maddalena, I.S.C. leg”. **State of Rio de Janeiro:** 73 specimens (4 ANIC; 67 CELC, including one dissected male; 2 CEMT) “Brasil: RJ, Seropédica, 2004, Grossi, P.C. leg.\ex. *Pycnoporus sanguineus*”; 12 specimens (CELC) “Brasil: RJ, Porciúncula, 30.x.2016, Pecci-Maddalena, I.S.C. & Folly, C. leg.\ex *Pycnoporus sanguineus*”; 12 specimens (CELC, including one dissected male) “Brasil: RJ, Rio das Ostras, Rebio União, 01.iii.2013, Aloquio, S. leg.\ex. *Pycnoporus sanguineus*”; 12 specimens (CELC, including one dissected male) “Brasil: RJ, Grussaí, 01.xi.2003, Souto, L.S. leg.\ex. *Pycnoporus sanguineus*”; 4 specimens (CELC, including one dissected male) “Brasil: RJ, Paraty, Ponta Negra, 5-6.iii.2011, Sandoval, V.E & Idrobo, C.J. leg.”. **State of São Paulo:** 3 specimens (2 ANIC; 1 CELC, dissected male) “Ilha da Victoria, S. Paulo, Brazil, Dec. 1963, Exped. Dep. Zool.\*Ceracis cornifer* 9 (Mellié) 202”; 1 specimen dissected (CELC) “Peruibe, SP, 28.xi a 01.xii.84\Exp. MZUSP em fungo\ *Ceracis ruficornis* Pic. Gen 1 [handwritten]”; 4 specimens (MZSP, including one dissected male) “Raiz da Serra, SP, 28.ix.1907, Luderwaldt\Dep. Zool. São Paulo 1247\SP”; 3 specimens (CELC) “Brasil: SP, São José dos Campos, Parque da cidade, 09.x.2004, leg S.S.P. Almeida & G.S.P. Almeida\ex. *Pycnoporus sanguineus*”. **State of Espírito Santo:** 10 specimens (CELC, including one dissected male) “Brasil: ES, Alto Bergamo, João Neiva, 11.v.2008, Furieri, leg.\ex. *Pycnoporus sanguineus*”; 23 specimens (2 NMNH; 2 ANIC; 19 CELC) “Brasil: ES, Santa Teresa, EBSL, 18-24.ix.2015, Pereira, M.R. leg., trilha atrás do alojamento\ex *Pycnoporus* sanguineus”; 3 specimens (CELC) “Brasil: ES, Santa Teresa, Reserva Biológica de Santa Lúcia, 03.iii.2003, Barreto, F.C.C & Furieri, K.S. leg.”; 10 specimens (CELC) “Brasil: ES, Santa Teresa, Rebio Augusto Ruschi, 17-19.vi.2013, Pereira, M.R. leg.\ex. *Pycnoporus sanguineus*”; 1 specimen (CELC) “Brasil: ES, Santa Teresa, EBSL, iii.2003, Furieri, K.S. leg.\ex. *Pycnoporus sanguineus*”; 1 specimen dissected (CELC) “Brasil: ES, Conceição da Barra, Rebio Córrego Grande, 14−19.xii.2005, Furieri, K.S., Loiola, G.R., Van de Koken, A.F. legs. [sic]”; 1 specimen dissected (CELC) “Brasil: ES, Conceição da Barra, Floresta Nacional do Rio Preto, 03.xii.2011, Araujo, L.S. leg.\Trans: 3, Parcela B, Fungo 1”; 2 specimens (CELC) “Brasil: ES, Conceição da Barra, Floresta Nacional do Rio Preto, 03.xii.2011, Araujo, L.S. leg.\Trans: 3, Parcela 0, Fungo 4”; 2 specimens (CELC) “Brasil: ES, Conceição da Barra, Floresta Nacional do Rio Preto, 03.xii.2011, Araujo, L.S. leg.\Trans: 1, Parcela 0, Fungo 1”; 1 specimen (CELC) “Brasil: ES, Conceição da Barra, Floresta Nacional do Rio Preto, 03.xii.2011, Araujo, L.S. leg.\Trans: 2, Parcela C, Fungo 1”; 1 specimen (CELC) “Brasil: ES, Conceição da Barra, Floresta Nacional do Rio Preto, 03.xii.2011, Araujo, L.S. leg.\Trans: 2, Parcela A, Fungo 5”; 1 specimen (CELC, dissected) “Brasil: ES, Aracruz, 04.i.2004, Furieri, K.S. leg.”. **State of Bahia:** 2 specimens (CELC, including one dissected male) “Brasil: BA, Porto Seguro, RPPN Pau Brasil, 4−7 Janeiro 2010, Chamorro, J. leg.\ex. *Pycnoporus sanguineus*”. **State of Mato Grosso do Sul:** 9 specimens (CELC, including one dissected male) “Brasil: MS, Paranhos, Chácara Santo Antônio, Pastagem, vi.2012, Puker, A. leg.\ex. *Pycnoporus sanguineus*”; 6 specimens (CELC, including one dissected male) “Brasil: MS, Campo Grande, Cerradinho UFMS, 27.v.2015, Chamorro, J. leg.\ex. *Pycnoporus sanguineus*”. **State of Paraná:** 1 specimen (DZUP) “ANTONINA –PR, Reserva Sapitanduva, BRASIL 27.I.1987, Lev. Ent. PROFAUPAR, LÂMPADA [printed]\DZUP 273631 [printed]”; 1 specimen (DZUP) “ ANTONINA –PR, Reserva Sapitanduva, BRASIL 30.III.1987, Lev. Ent. PROFAUPAR, LÂMPADA [printed]\DZUP 273654 [printed]”; 1 specimen (DZUP) “GUARAPUAVA – PARANÁ, Est. Águas Sta. Clara, BRASIL 27.I.1987, Lev. Ent. PROFAUPAR, LÂMPADA [printed]\DZUP 273632”. **State of Santa Catarina:** 2 specimens (MNRJ) “Brasilien, Nova Teutonia, 27°11 B, 52°23’ L, Fritz Plaumann [printed]”; 3 specimens (MNRJ) “Brasilien, Nova Teutonia, 27°11 B, 52°23’ L, Fritz Plaumann [printed]”; 3 specimens (MNRJ) “9 [handwritten], 1936 [“6” handwritten and “193” printed], Brasilien, Nova Teutonia, 27°11 B, 52°23’ L, Fritz Plaumann [printed]”; 24 specimens (CELC, including one dissected male) “Brasil, SC, Urubici, Estrada p/Serra do Corvo Branco em placa, 06.iii.2011, Grossi & Perigotto legs. [sic]\28°02’53’’S, 49°23’06’’W, 1022 m\ex. *Pycnoporus sanguineus*”; 30 specimens (CELC) “Brasil, SC, Urubici, Estrada p/Serra do Corvo Branco em placa, 06.iii.2011, Grossi & Perigotto legs.\28°02’53’’S, 49°23’06’’W, 1000 m\ex. *Pycnoporus sanguineus*”. **State of Rio Grande do Sul:** 3 specimens (CELC, including one dissected male) “Brasil: RS, São Francisco de Paula, Flona SFP, 2006, Graf, L.V. leg.\ex. *Pycnoporus sanguineus*; 15 specimens (CELC) “Brasil: RS, São Francisco de Paula, Flona SFP, xii. 2006, Graf, L.V. leg.\Fungo 1970, sp 29”; 51 specimens (23 CELC; 28 MCNZ) “Brasil: S. Fran. Paula, RS (FLONA) IX. 2006 L. V. Graf col.\Em *Pycnoporus sanguineus*”; 41 specimens (MCNZ) “Brasil: RS, Santa Tereza, 03.IX.2015, 26.X.2015, 10.II.2016, 18.III.2016, Mezzomo, A.G leg. **ARGENTINA:** 12 specimens (CELC, including one dissected male) “Argentina, Misiones Prov. nr. Wanda, 25°58’S, 54°35’W, 22.x.2007 S. Neser\Ex. Orange bracket fungus on old pine logs\National coll. of insects\Pretoria, South Africa”; 3 specimens (2 ANIC; 1 CELC, dissected male) “Arg: Salta Capital, July, 1971, L.A. Stange, Lot 14\ *Pycnoporus cinnabarinus*”; 2 specimens (1 ANIC; 1 CELC) “Arg. Tucuman, Rio Urueña, nr, Salta, Border, Dec 1970\L.A. Stange Lot 4(2)\*Pycnoporus cinnabarinus*”; 2 specimens (1 ANIC; 1 CELC) “Arg. Tucuman, Tafi del Valle, xii.17.1971\L.A. Stange Lot 15”; 2 specimens (1 CELC, dissected male) “Tucuman, Arg.v.1926, R.C. Shannon\ex. *Polyporus sanguineus*\Ceracis (9) sp 448”; 2 specimens (1 ANIC; 1 CELC) Tucuman“Arg. Feb. ’29, R.C. Shannon\ex. *Polyporus sanguineus*”; 4 specimens (1 NMNH; 2ANIC; 1 CELC) “Tucuman, Argentina, L. Castillon, Coll. J.F. Lawrence, Lot. 2007\ex. *Polyporus sanguineus*, ex U.S.D.A Herbaria”. **URUGUAY:** 7 specimens (5 ANIC; 2 CELC, including one dissected male) “Montevideo, Uruguay, F. Felippone, J.F. Lawrence, Lot. 2025\ex. *Trametes hispida*, ex U.S.D.A Herbaria”. **MEXICO:** 6 specimens (4 ANIC; 2 CELC, including one dissected male) “Cer furcifer gr. Det. J.F. Lawrence 19\Mazatlan Crotch”. **UNITED STATES:** 3 specimens (2 ANIC; 1 CELC, dissected male) “4 mi. SE Lake Placid Highlands County Florida, June 30, 1965\J.F. Lawrence Lot 1532\*Polyporus sanguineus*”.

**Remarks.** The names here synonymized with *C. cornifer* are of species described largely on differences in shape and length of the frontoclypeal horn of males and in dorsal coloration of individuals. However, a careful examination and comparison of specimens, especially of sclerites of male terminalia, showed that these differences represent intraspecific variation. In all examined populations of *C. cornifer*, the male frontoclypeal horn is extremely variable in length and even in shape. There are small male morphs with the apex of the frontoclypeal horn slightly bifurcated or truncated, intermediate morphs, and large morphs like the lectotype of *C. cornifer* (Figure 5A−H). Small males with a small frontoclypeal horn slightly bifurcated at apex are relatively common (e.g., see the small horns in Figure 5C−F) but bifurcation in large males with large horns is rare (one case shown in Figure 5H). Some specimens from Ubá (MG, Brazil) and Grussaí (RJ, Brazil) have the lateral contours of pronotum angulate and the anterior edge forms two prominent lobes. The coloration in both sexes varies from yellowish-brown or reddish-brown, even in individuals from a single locality, to black (especially specimens of the southern tip of the distributional range) (Figure 6A−C). The male specimen from Florida (EUA), previously identified as *C. monocerus*, was not measured due to its poor condition, except for its TL. Nevertheless, TL (1.18 mm), body shape and anatomy of male abdominal terminalia show that it is like the large morphs of *C. cornifer* from South American localities and is considered here to be a synonym of *C. cornifer*. *Ceracis simplicicornis* (Pic), also proposed as a new synonym of *C. cornifer*, was described as “nigro-piceus” (Figure 6D) but we observed that it is just a colour variation in populations from Argentina and southern Brazil. The type-locality of both *C. cylindricus*, of which the type is lost, and *C. simplicicornis* is Buenos Aires. Having examined many specimens from southern South America, we concluded that the diagnostic features in the descriptions of both *C. cylindricus* and *C. simplicicornis* represent intraspecific variation of *C. cornifer. Ceracis unicornis* is an interesting case, before the revision of North American *Ceracis* by Lawrence [3], species described as *Ceracis* were those with eight antennomeres, while species described as *Ennearthron* were those with nine antennomres. Although *C. unicornis* was described as a *Ceracis*, it has nine antennomeres, which was first noted by Lawrence [3] and confirmed by us. *Ceracis unicornis* was described as “nigro-piceus”, a colour variation common in other populations of *C. cornifer*, as pointed out above; it is another intraspecific variation and is here synonymized with it *C. cornifer*. The examined specimens from Campo Grande (MS, Brazil) resemble *C. ruficornis*, with the horn apex slightly bifurcated, body uniformly shiny black and legs with a somewhat amber colour, but a more careful examination and dissection of males allowed reliable identification as *C. cornifer*. Examined specimens from Paranhos (MS, Brazil) are also black (Figure 6, VII). Aside from these colour variations, teneral forms, which are light colored, occur in all populations. Male terminalia of all dissected specimens of *C. cornifer*, including those from populations previously identified as *C. monocerus* and *C. simplicicornis*, are extremely similar, mainly in the shape of penis and sternite VIII. In the male specimen from Florida (EUA), the emargination on the outer portion of each lobe is more prominent than in specimens from Brazil. All observed variation in male terminalia was weak or inconsistent in a population or between populations, and was not consistent with the observed variation in body colour, and length and shape of the male frontoclypeal horn. Therefore no support could be found for maintaining *C. cylindricus*, *C. monocerus*, *C. simplicicornis* and *C. unicornis* as separate species.

**Distribution.** *Ceracis cornifer* is widely distributed in the southern, southeastern and southwestern Neotropical region, without records from the central and northern South America, but occurring in southwestern Mexico, in a few Caribbean islands and southern USA (Figure 2). This species is syntopic with *C. furcifer* in Paranhos (southwestern Brazil) and “Serra do Cipó” (locality of Jaboticatubas, southern Brazil). In these two samples, *C. cornifer* and *C. furcifer* were found living in the same basidiomes of *P. sanguineus*. *Ceracis cornifer* and *C. ruficornis* are also sympatric in three localities in southeastern Brazil (Paraty, Peruíbe and Ilha da Victória) (Figure 2). 

#### 3.2.2. *Ceracis furcifer* Mellié, 1849

**Figure 7:**
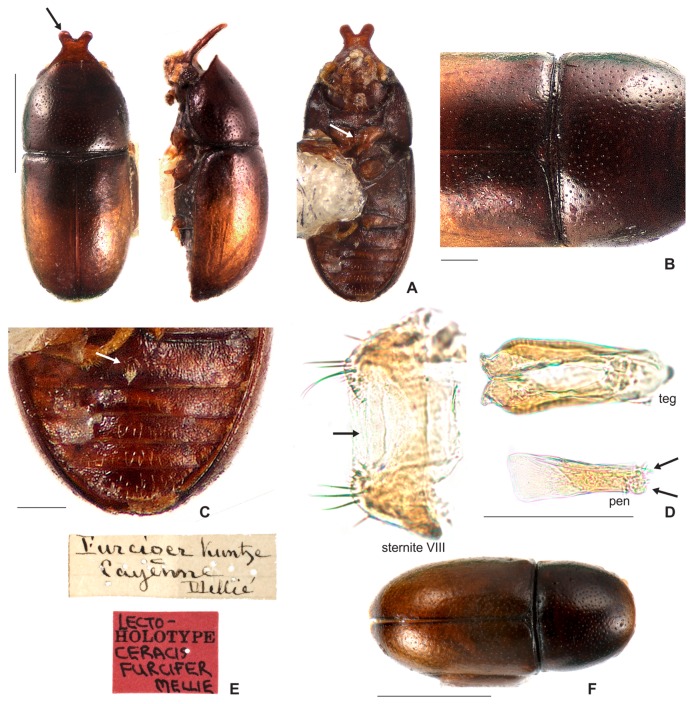
***Ceracis furcifer* Mellié**. **A−C** Male lectotype, (**A**) dorsal, lateral and ventral views, respectively. Horn apex with two conspicuous rounded lobes (black arrow); thin prosternal process (white arrow), (**B**) pronotal punctation, (**C**) first abdominal ventrite with a sex patch at centre (white arrow), (**D**) male terminalia in a topotype, showing sternite VIII with a deep concave emargination at middle (black arrow), tegmen (teg), penis (pen) with the basal edge blunt (black arrows), (**E**) labels of the lectotype deposited in the Muséum d’Histoire Naturelle, MHNG (Géneve, Switzerland), (**F**) female plesiotype from Manaus (AM, Brazil). Scale bars: A = 0.5 mm, B−D = 0.1 mm, F = 0.5 mm.

**Figure 8:**
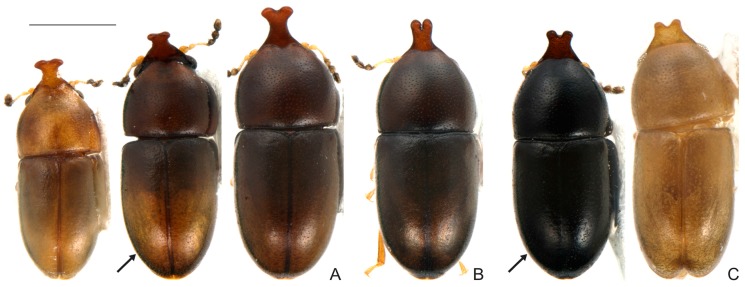
**Colour variation in specimens of *Ceracis furcifer* Mellié**. (**A**) Colour variation among individuals from a single locality (Paranhos, MS, Brazil), arrow showing specimen “half brown, half dark-brown”. (**B**) A specimen from Mambaí (GO, Brazil) showing an intermediate colour in comparison to specimens shown in A and C. (**C**) A teneral (right) and homogeneously black individual (left, black arrow) from Araguaína (TO, Brazil). Scale bar: 0.5 mm.

Figure 1D; Figure 5I,J; Figure 7A–F; Figure 8 and Figure 11.4.

*Ceracis furcifer* Mellié, 1849: 379, pl. 4, Fig24. Type locality: Cayenne, French Guyana [1]; Gorham: 1886:359 [44] {taxonomic notes and distribution}; 1898: 332 [12] {taxonomic notes}; Blackwelder 1945: 550 [14] {distribution}; Lawrence 1967: 97 [3] {taxonomic status}; 1973: 202 [21] {feeding habit}; Abdullah 1973: 199 [15] {taxonomic status and distribution}; Mueller 2001: 317 [45] {taxonomic notes and distribution}; Lopes-Andrade 2002: 6 [43] {taxonomic notes}; Gumier-Costa et al., 2003: 359 [9] {host fungus and distribution}; Graf-Peters 2011: 558 [10] {feeding habitat}; Antunes-Carvalho and Lopes-Andrade 2011: 61 [5] {taxonomic notes}; Pecci-Maddalena 2014: 482 [7] {taxonomic notes}; Peck 2015: 148 {distribution} [16]; Lopes-Andrade and Grebennikov 2015: 476 [34] {molecular data}. 

*Ceracis semipallidus* Pic, 1922: 3. Lawrence 1967: {junior synonym}. Type locality: Guadeloupe, Caribbean [46].

**Diagnosis.** The apex of the frontoclypeal horn of males is elongate and emarginated at middle, forming two conspicuous lobes with rounded apex; the lateral contours of horn lack inflection near base, but in the largest morphs the horn is conspicuously narrowed at middle. The antennal funicle has three antennomeres, in which the first is equal or longer than the next three antennomeres together. The apical portion of tegmen has a short inner emargination, which is less than one half of the tegmen length; the lateral edges have an enlargement before apex, and the outer apical portion of each lobe bears a prominent inflection. 

**Redescription. Lectotype, here designated (Figure 7A−C,E).** Adult male apparently fully pigmented. Measurements (in mm): TL 1.2, PL 0.44, PW 0.543, EL 0.76, EW 0.56, GD 0.47. Ratios: PL/PW 0.81, EL/EW 1.36, EL/PL 1.73, GD/EW 0.84, TL/EW 2.14. **Body** glabrous, elongate, subcylindrical; frontoclypeal horn mostly reddish-brown; dorsum, from apex of pronotum to elytral disc, dark reddish-brown; apical half of elytra, reddish-brown; venter mostly dark reddish-brown, except for the reddish-brown abdominal ventrites; basal antennomeres, funicle, mouthparts and legs yellowish-brown, but antennal club reddish-brown. **Head** barely visible from above; area immediately above horn base concave, glabrous and sparsely punctate; frontoclypeal ridge strongly produced forming a long horn directed upwardly, laminate in lateral view (in mm: length 0.3, basal width 0.3); horn apex elongate, emarginated at middle, forming two conspicuous rounded lobes (Figure 7A, black arrow; Figure 11.4, big black arrow); lateral contours of horn without inflection near base, but conspicuously narrowed at middle (Figure 11.4, small black arrow). **Antennae** (right antennae measured) with FL 0.08 CL 0.14, CL/FL 1.75, length of antennomeres 1−8 (in mm) as follows: 0.06, 0.04, 0.04, 0.02, 0.02, 0.03, 0.05, 0.06; sensillifers barely visible. **Eyes** coarsely faceted, with minute slender yellowish in intersections of ommatidia; GW 0,11 mm. **Pronotum** subquadrate, with anterior portion produced forward, concealing head when seen from above; anterior edge slightly emarginated at middle, forming two small lobes; sides narrow, not visible from above; posterior edge sublinear; punctation fine, single, uniform and regularly distributed (Figure 7B); distance between punctures about 2 to 3 puncture-widths; each puncture bearing a yellowish decumbent minute setae, barely visible even at a magnification of 150x; interspaces microreticulate. **Scutellum** small, subtriangular, with few punctures, each one bearing a short, fine decumbent setae; BW 0.08 mm and SL 0.05 mm. **Elytra** about 1.7x as long as pronotum; sides subparallel at the basal two-thirds, then abruptly converging toward apex; punctation single, finer than pronotal punctation; humeral calli conspicuous. **Metathoracic wings** developed, apparently functional. **Prosternum** in front of coxae shallowly concave; prosternal process thin (Figure 7C, white arrow), similar to *Cer. cornifer* species (Figure 4A) and parallel-sided, almost as long as coxae and projected below prosternal disc. **Hypomera** biconcave, subglabrous, with lateral contours emarginated. **Pro-, meso- and metathoracic tibiae** absent in the lectotype, but other specimens examined have the pattern found in species of the *furcifer* group (see the diagnosis of the *furcifer* group). **Metaventrite** microreticulate, subglabrous and moderately convex. **Abdominal ventrites** microreticulate; punctures shallow; vestiture of scattered slender setae, longer than those on dorsum; length of ventrites I−V (in mm, from base to apex of each ventrite at the longitudinal midline): 0.14, 0.06, 0.05, 0.05, 0.08. First abdominal ventrite bearing a circular, marginated, pubescent sex patch at centre (Figure 7C, white arrow), similar to *Cer. cornifer* (Figure 4B). **Male abdominal terminalia** (Figure 7D in topotype) with posterior edge of **sternite VIII** bearing a deep, concave, mesal emargination (Figure 7D, sternite VIII, black arrow); posterior corners sclerotised and rounded, bearing bristles; median portion membranous; lateral edges diverging from posterior to anterior portion; anterior edge sublinear. **Tegmen** (Figure 7D, teg and 11.4, teg) with apical portion bearing a short inner emargination, less than half total length; lateral edges enlarged near apex (Figure 11.4, teg, small red arrows), with a prominent inflection at the external apical portion of the lobes (Figure 11.4, teg, big red arrows). **Penis** elongate, subcylindrical with blunt basal edge (Figure 7D, pen, arrows); sclerotised and subparallel from base to about middle, then expanding to a membranous apical portion. 

**Females** (Figure 7F). Like males, but abdominal sex patch absent and frontoclypeal ridge devoid of horn. They resemble females of *C. cornifer*, but in the latter each antenna has nine antennomeres instead of eight. 

**Variation.** Males, measurements in mm (n = 32, included the lectotype) TL 1.02−1.35 (1.16 ± 0.09); PL 0.37−0.51 (0.43 ± 0.04); PW 0.44−0.6 (0.52 ± 0.04); EL 0.6−0.88 (0.73 ± 0.07); EW 0.47−0.61 (0.55 ± 0.04); GD 0.4−0.56 (0.47 ± 0.04); HL 0.05−0.38 (0.23 ± 0.07). Ratios: PL/PW 0.76−0.92 (0.83 ± 0.03); EL/EW 1.14−1.52 (1.34 ± 0.1); EL/PL 1.33−1.95 (1.7 ± 0.15); GD/EW 0.78−0.96 (0.86 ± 0.04); TL/EW 1.93−2.33 (2.13 ± 0.1). Females, measurements in mm (n = 19) TL 1.02−1.32 (1.17 ± 0.08); PL 0.37−0.5 (0.43 ± 0.03); PW 0.42−0.55 (0.48 ± 0.03); EL 0.65−0.85 (0.74 ± 0.05); EW 0.44−0.58 (0.52 ± 0.03); GD 0.4−0.52 (0.46 ± 0.03). Ratios: PL/PW 0.83−1 (0.89 ± 0.05); EL/EW 1.28−1.54 (1.42 ± 0.06); EL/PL 1.47−1.88 (1.72 ± 0.1); GD/EW 0.83−0.96 (0.88 ± 0.03); TL/EW 2.14−2.4 (2.25 ± 0.06). Specimens of the following localities were measured: **BRAZIL:** Mato Grosso do Sul (Paranhos); Góias (Mambaí); Pará (Marabá); Tocantins (Araguaína); Amazonas (Manaus). **COLOMBIA:** Casanare (San Luis de Palenque). **COSTA RICA:** Turrialba. **MEXICO:** Veracruz (Coatzacoalcos) and **FRENCH GUYANA:** Cayenne.

**Material examined. Lectotype male, here designated (MHNG) (Figure 7A)** “*Furcifer* Kuntze Cayenne Mellié [handwritten]\[red label] Lectotype *Ceracis furcifer* Mellie [handwritten]”. **Historical Material:** 1 specimen (MHNG) “*Furcifer* Kuntze Cayenne Mellié [handwritten]\[yellow label] PARALECTOTYPE [printed] *Ceracis furcifer* Mellié [handwritten]”; 1 specimen (MHNG) “*Coll. Melly* [printed]\[yellow label] PARALECTOTYPE [printed] *Ceracis furcifer* Mellié [handwritten]”; 2 specimens (MHNG, including one dissected male) “*Furcifer* Kuntze Perou Mellié [handwritten]\[yellow label] PARALECTOTYPE [printed] *Ceracis furcifer* Mellié [handwritten]”; 6 specimens (MHNG) “*Coll. Melly* [printed]\[yellow label] PARALECTOTYPE [printed] *Ceracis furcifer* Mellié [handwritten]; 2 specimens (MHNG, including one dissected male) “*Furcifer* Kuntze mexique Geory Mellié [handwritten]”; 1 specimen (MHNG) “*furcifer* Kuntze Suriname Geory Mellié [handwritten] [specimens dandified, without head and pronotum]”; 1 dissected specimen (MFN) “[green label] Hist. Coll. (Coleoptera) Nr. 53172 (3.Ex.) Cis furcifer Kunze Zool. Mus. Berlin [printed]”; 1 dissected specimen (MNHN) “[green label] MUSEUM PARIS GUYANE FRANÇAISE St-Laurent du Maroni E. Le moult 1911?”; *Ceracis semipallidus*, lectotype male, here designated, dissected (MNHN) “[red label] LECTOTYPE [printed] *Ceracis semipallidus* Pic [handwritten]\*Ceracis semipallidus* mp? [handwritten]\Ciside n°241.3[handwritten]\Guadeloup [printed]\Ex. M. Pic Collection Générale (ex. Anceyiete) Exotique [handwritten]”. **BRAZIL. State of Minas Gerais:** 40 specimens (2 CERPE; 38 CELC) “Brazil: MG, PN Serra do Cipó, próx. à Cachoeira da Farofa, -19.385724, -43. 585808, 11.v.2016, 758 m, Em grande tronco podre caído, leg. J. Chaul & E. Epifânio\ex *Pycnoporus sanguineus*; **State of Amazonas:** 30 specimens (2 CAMB; 2 CNCI; 26 CELC, including one dissected male) “Brasil: AM, Manaus, Campinara, 12.vii.2011, Pereira, M.R. leg.\ex. *Pycnoporus sanguineus*”; 28 specimens (2 FMNH; 2 CAMB; 2 CMN; 22 CELC, including one dissected male) “Brasil: AM, Manaus, Tarumã-Mirim Rio Negro, 10.vi.2011, Pereira, M.R. leg.\ex. *Pycnoporus sanguineus*”. **State of Pará:** 30 specimens (CELC, including one dissected male) “Brasil: PA, Marabá, Reserva Biológica de Tapirapé, pastagem, 2003, Gumier-Costa, F. leg.”; 15 specimens (CELC) “Brasil: PA, Marabá, Reserva Biológica de Tapirapé, Amazônia Legal, 04.xii.2003, Gumier-Costa, F. leg.\Amostra extra n°09, Borda”; 12 specimens (10 CELC; 2 CEMT) “Brasil: PA, Marabá, Reserva Biológica de Tapirapé, Amazônia Legal, 03.xii.2003, Gumier-Costa, F. leg.\AMOSTRA n°59, Pasto”; 6 specimens (CELC) “Brasil: PA, Marabá, Reserva Biológica de Tapirapé, Amazônia Legal, 04.xii.2003, Gumier-Costa, F. leg.\AMOSTRA n°72, Pasto”. **State of Góias:** 10 specimens (CELC, including one dissected male) “Brasil: GO, Mambaí, Fronteira GO/BA, maio 2012, Nilber leg.”; 7 specimens (CELC, including one dissected male). **State of Mato Grosso do Sul** “Brasil: MS, Paranhos, Chácara Santo Antônio, Pastagem, vi.2012, Puker, A. leg.\ex. *Pycnoporus sanguineus*”; 1 specimen (CELC) “Brasil: MS, Paranhos, Chácara Santo Antônio, Pastagem, vi.2012, Puker, A. leg.”. **State of Tocantins:** 20 specimens (CELC) “Brasil: TO, Araguaína, Campos UFT/ EMUZ, vi.2015, Sandoval-Gómez, V.E. leg.\ex. *Pycnoporus sanguineus*.”; **COLOMBIA:** 20 specimens (CELC, including one dissected male) “Colômbia, Casanare San Luis de Palenque, 260 m, 30.i.2010, Contreras, J. L leg.\ex. *Pycnoporus sanguineus*”; 4 specimens (3 ANIC; 1 CELC, dissected male) “Colômbia: Valle Rio Jamundi entre Cali y Jamundi, 1000 m\slipt corn”. **PANAMÁ:** 4 specimens (3 FMNH; 1 CELC including one dissected male) “Madden Dam, Canal Zone, vii-18-1969, J.F. Lawrence\Lot 2901\ex. *Daedalea elegans*”; 5 specimens (4 FMNH; 1 CELC, dissected male) “R. Panamá: Almirante, 1959, H.S. Dybas, FMNH (HD) # 59-154\*Polyporus sanguineus*”. **JAMAICA:** 1 specimen dissected (ANIC) “Kingston, Jamaica, W.I., A.H. Ritchie, J.F. Lawrence, Lot 1991\ex. *Polyporus maximus* ex USDA herbaria\Ceracis furcifer (8) mellié 203”. **ANTIGUA:** 2 specimens (1 ANIC; 1 CELC, dissected male) “Gaynor’s Gut, Antigua, B.W.I, IX-11.65, A.P. Laska\J. F. Lawrence, Lot 1598\ex. *Polyporus sanguineus*”. **COSTA RICA:** 3 specimens (2 ANIC; 1 CELC, dissected male) “Turrialba, Costa Rica, ix-5-66\JF Lawrence Lot 1851\Robin Andrews Collector\ex. *Daedalea elegans*”; 4 specimens (3 ANIC; 1 CELC, dissected male) “Smi. S.W. Cañas Guanacaste, Costa Rica, Feb. 3-12.1967\J.F. Lawrence Lot.2153\ Robin Andrews Coll.\ex. *Polyporus sanguineus*”. **MEXICO:** 1 dissected specimen (ANIC) “Mex: Veracruz, Coatzacoalcos\mi.S.vii.10.63\ J. Doyen collector”.

**Remarks.** We dissected the lectoypes of *C. furcifer* and *C. semipallidus*, compared their male genitalia and confirmed the synonymy proposed by Lawrence [3]. The shape of the frontoclypeal horn in males of *C. furcifer* is variable. However, its apex is usually bifurcated in small to large morphs (Figure 5I,J). In small morphs, this bifurcation may be very prominent (Figure 5J). The occurrence of more than one body colour among individuals from the same population is common (Figure 8A) and the coloration “half brown, half dark-brown” seems to be one of the most common (Figure 8A, arrow). There are populations in which individuals are homogeneously black (Figure 8C, arrow), but other intermediate colorations can also occur (e.g., Figure 8B). The lobes of tegmen are rounded and comparatively larger in some males (e.g., specimens from Manaus, Brazil); in other cases, these lobes are conspicuously angulate (e.g., in a specimen from Antigua, Caribbean Sea).

**Distribution.** *Ceracis furcifer* occurs overwhelmingly incentral and northern South America, extending its range throughout the Antilles until Mexico (Figure 2). This species is syntopic with *C. cornifer* in Paranhos (southwestern Brazil) and “Serra do Cipó” (locality of Jaboticatubas, southern Brazil). In these two samples, *C. furcifer* and *C. cornifer* were found living in the same basidiomes of *P. sanguineus*. *Ceracis furcifer* is also syntopic with *C. hastifer* in San Luis de Palenque (Colombia) and these two species were also found living in the same basidiomes of *P. sanguineus*.

#### 3.2.3. *Ceracis hastifer* (Mellié, 1849)

**Figure 9:**
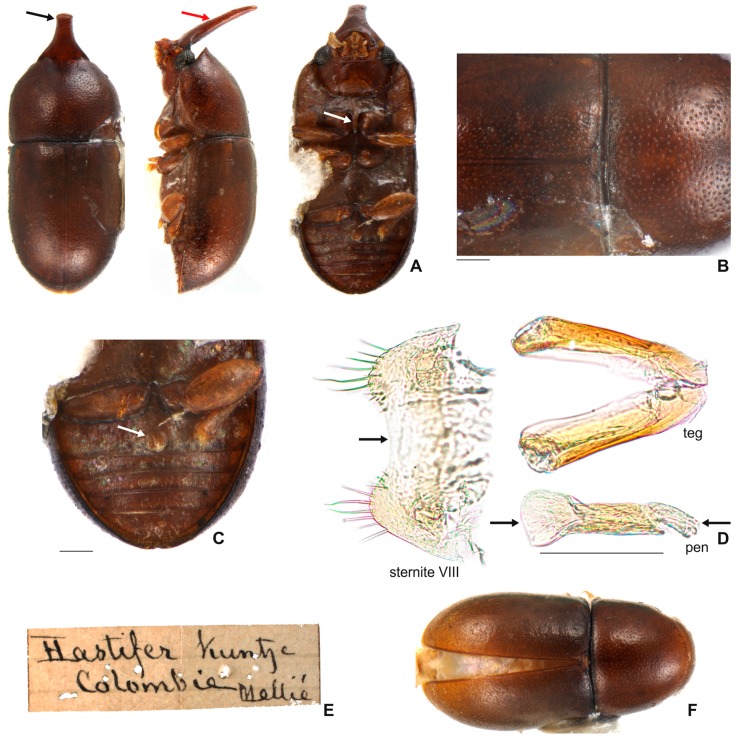
***Ceracis hastifer* Mellié. A−C** Male lectotype, (**A**) dorsal, lateral and ventral views, respectively. Frontoclypeal ridge strongly produced forming a long and narrow upward directed median horn (black arrow); laminate in lateral view (red arrow); prosternal process thin (white arrow), (**B**) pronotal punctation, (**C**) first abdominal ventrite with a sex patch at the centre (white arrow), (**D**) male terminalia in a paralectotype, showing sternite VIII with a deep concave emargination at the middle of the posterior edge (black arrow); tegmen (teg), penis (pen) with membranous apical and basal portion (arrow), (**E**) labels of the lectotype deposited in the Muséum d’Histoire Naturelle, MHNG (Géneve, Switzerland), (**F**) female plesiotype from Peru. Scale bars: A = 0.5 mm, B−D = 0.1 mm, F = 0.5 mm.

Figure 5K,L; Figure 9A-F and Figure 11.5’ 

*Ennearthron hastiferum* Mellié, 1849: 370, pl. 4, Figure 17. Type locality: Colombie [1]; Pic: 1916: 20 [13] {taxonomic notes}; Blackwelder 1945: 549 [14] {distribution}; Lawrence 1967: 97[3] {taxonomic status}; Abdullah 1973: 199 [15] {taxonomic status and distribution}; Mueller et al., 2001: 318 [45] {taxonomic notes}.

*Ceracis hastifer* (Mellié, 1849): Lawrence 1967: 99 [3] {taxonomic notes}; Abdullah 1973: 199 [15] {taxonomic status and distribution}; Mueller et al., 2001: 318 [45] {taxonomic notes}; Antunes-Carvalho and Lopes-Andrade 2011: 61 [5] {taxonomic notes}; Pecci-Maddalena 2014: 486 [7] {taxonomic notes}. 

**Diagnosis.** The base of the frontoclypeal horn in males does not have lateral inflection and the horn is expanded until one third of its length. The antennal funicle has four antennomeres, the first being shorter than the following three antennomeres together. The anterior portion of hypomera has the outer edge broadly rounded. The lateral edges of tegmen are almost linear, without inflection; the apical portion bears a deep inner emargination at least two-thirds the length of tegmen, forming two long lateral lobes; the outer apical edges of lobes are apparently devoid of emargination.

**Redescription. Lectotype, here designated (Figure 9A−C,E).** Adult male apparently fully pigmented; lacking antennae. Measurements (in mm): TL 1.39, PL 0.49, PW 0.69, EL 0.9, EW 0.7, GD 0.56. Ratios: PL/PW 0.71, EL/EW 1.3, EL/PL 1.84, GD/EW 0.8, TL/EW 1.94. **Body** glabrous, elongate. Dorsum mostly dark reddish-brown; venter mostly reddish-brown, except for yellowish-brown hypomera, legs and mouthparts. **Head** barely visible from above; area immediately above horn base concave, glabrous, sparsely punctate. Frontoclypeal ridge strongly produced forming a long, narrow median horn directed upwardly (in mm: length 0.5, basal width 0.32) (Figure 9A, black arrow), laminate in lateral view (Figure 9A, red arrow) and bearing several minute, sparse setae; horn largest at base and tapering until its basal one-third (Figure 11.5’, big black arrow), devoid of lateral inflection, with apex rounded, subtruncate. **Antennae** (left antennae measured in another specimen) FL 0.09 mm, CL 0.16 mm, CL/FL 1.8, length of antennomeres 1−9 (in mm) as follows: 0.06, 0.04, 0.03, 0.03, 0.02, 0.01, 0.04,0.05,0.07; sensillifers barely visible. **Eyes** coarsely faceted, with minute slender yellowish setae in intersections of ommatidia (barely visible even at a magnification of 150x), GW 0.13 mm. **Pronotum** with anterior portion produced forward, concealing head when seen from above; anterior edge shallowly emarginated, forming two short and acute frontolateral lobes; sides narrow, not visible from above; posterior edge sublinear, punctation single, uniform and regularly distributed (Figure 9B), with microreticulate interspaces; distance between punctures from about 1 to 3 puncture-widths; each puncture bearing a yellowish decumbent minute setae, barely visible even a magnification of 150x. **Scutellum** small, subtriangular, with few punctures, each one bearing a short, fine, decumbent seta; BW 0.09 mm and SL 0.04 mm. **Elytra** about 1.84x as long as pronotum; sides subparallel at the basal two-thirds, then abruptly converging toward apex; punctation single, finer than pronotal punctation; humeral calli conspicuous. **Metathoracic wings** developed, apparently functional. **Prosternum** in front of coxae shallowly biconcave; prosternal process thin (Figure 9A, white arrow), similar to *Cer. cornifer* species (Figure 4A) and parallel-sided, almost as long as coxae and projected below prosternal disc. **Hypomera** with outer edge of anterior portion broadly rounded. (Figure 11.5’, small black arrow). **Pro-, meso- and metathoracic tibiae** similar to the ones of other *furcifer* group species (see the diagnosis of the *furcifer* group). **Metaventrite** moderately convex, subglabrous, microreticulate, bearing scattered slender setae; discrimen not discernible. **Abdominal ventrites** microreticulate, bearing shallow punctures and scattered slender setae longer than those on dorsum; length of the ventrites I−V (in mm, from base to apex of each ventrite at the longitudinal midline) 0.19, 0.05, 0.05, 0.06, 0.08. First abdominal ventrite bearing a circular, marginated, pubescent sex patch at the centre (Figure 9C, arrow), similar to *Cer. cornifer* species (Figure 4B), with a transverse diameter of 0.06 mm. **Male abdominal terminalia** (in a paralectotype) (Figure 9D) with the posterior edge of **sternite VIII** bearing a deep, concave, mesal emargination (Figure 8D, sternite VIII, arrow); posterior corners rounded, sclerotised, bearing bristles; disc membranous; lateral edges diverging from posterior to anterior portion; anterior edge slightly biconcave. **Tegmen** (Figure 9D, teg and 11.5’, teg) with deep inner emargination at the apical portion, about two-thirds the length of tegmen (Figure 11.5’, teg, red dashed line); lateral edges almost linear, without inflections; outer edges near apex devoid of emargination. **Penis** elongate, subcylindrical, basal and apical portions membranous (Figure 9D, pen, arrows); disc sclerotised; apical portion enlarged. 

**Females** (Figure 9F). Similar to males, except for being devoid of abdominal sex patch and horn at the frontoclypeal ridge. They mostly resemble females of *C. cornifer*, but are usually larger.

**Variation.** Males, measurements in mm (n = 6, included the lectotype) TL 1.14−1.46 (1.33 ± 0.1); PL 0.43−0.52 (0.47 ± 0.03); PW 0.58−0.69 (0.64 ± 0.03); EL 0.69−0.94 (0.86 ± 0.08); EW 0.6−0.7 (0.65 ± 0.04); GD 0.46−0.56 (0.52 ± 0.04); HL 0.27−0.51 (0.41 ± 0.08). Ratios: PL/PW 0.68−0.78 (0.73 ± 0.03); EL/EW 1.15−1.53 (1.33 ± 0.12); EL/PL 1.53−2.14 (1.84 ± 0.18); GD/EW 0.7−0.85 (0.8 ± 0.05); TL/EW 1.9−2.25 (2.05 ± 1.3). Females, measurements in mm (n = 3) TL 1.27−1.46 (1.35 ± 0.08); PL 0.46−0.57 (0.51 ± 0.04); PW 0.58−0.66 (0.6 ± 0.04); EL 0.77−0.89 (0.85 ± 0.05); EW 0.57−0.69 (0.62 ± 0.05); GD 0.5−0.54 (0.52 ± 0.02). Ratios: PL/PW 0.79−0.86 (0.84 ± 0.03); EL/EW 1.28−1.54 (1.37 ± 0.12); EL/PL 1.54−1.91 (1.67 ± 0.17); GD/EW 0.78−0.89 (0.84 ± 0.04); TL/EW 2.11−2.35 (2.19 ± 0.11). We measured all specimens that were in good conditions. 

**Material examined. Lectotype male, here designated(MHNG) (Figure 9A)** “*Hastifer* Kuntze Colombie Mellié, Melly Coll. [handwritten]\[yellow label] LECTOTYPE [printed] *Ennearthron hastiferum* Mellié [handwritten]\[white label] GEN [printed]”. **Historical Material:** One dissected male (MHNG) “*Hastifer* Kuntze Colombie Mellié [handwritten]\[yellow label] PARALECTOTYPE [printed] *Ennearthron hastiferum* Mellié [handwritten]”; 1 specimen (MHNG) “*Coll. Melly* [printed]\[yellow label] PARALECTOTYPE [printed] *Ennearthron hastiferum* Mellié [handwritten]”; 1 specimen (MHNG) “*Coll. Melly* [printed]\[yellow label] PARALECTOTYPE [printed] *Ennearthron hastiferum* Mellié [handwritten]”; 2 specimens (MHNG, including one dissected male) “*Hastifer* Kuntze Perou ?Mellié [handwritten]”. **COLOMBIA:** 1 dissected specimen (CELC) “Colômbia, Casanare San Luis de Palenque\1247965-1092026, 290.9 m, 30.i.2010, Contreras, J. L. leg.\ex. *Pycnoporus sanguineus*”; 1 dissected specimen (MFN) “\[blue label] Columb Moritz [handwritten]\[red label] Hist.-Coll. (Coleoptera) Nr. 53171 (4. Ex.) Cis hastifer Kunze Peru – Columb. Zool. Mus. Berlin [printed]. **PERU:** 1 specimen (SNSD) “Peru, Coll. Maerkel. [printed]\Staatl. Museum für Tierkunde Dresden [printed]”; 1 specimen (SNSD) “Peru, Coll. Maerkel. [printed]\Staatl. Museum für Tierkunde Dresden [printed]\Cis hastifer K Z ♂♀ Peru [handwritten]”; 4 specimens (3 NMNH; 1 CELC, dissected male) “Peru, Intercep Miami Florida 14576\ oct 19, 1960 ex Polyporid”. **BRAZIL:** 15 specimens (CELC) “Brasil: PA, Marabá, Reserva Biológica de Tapirapé, Amazônia Legal, 04.xii.2003, Gumier-Costa, F. leg.\Amostra extra n°09, Borda”; 1 dissected specimen (MPEG) “Brasil: AM, Tapurucuara, Rio-Negro, 1.2.1963, M.P.E. Goeldi, J & B. Bechyné”. 

**Doubtfully included.** 3 specimens (2 ANIC; 1 CELC, dissected male) “São Roque (Mato Dentro) S. Paulo, Brazil, xi-1963”\N. Papavero, Coll.”.

**Remarks.** We had few specimens in-hand, from a few localities. However, we observed important variation: the frontoclypeal horn of the male from Colombia (Casanare, San Luis de Palenque) has an inflection about four-fifths its length and a square shaped apex. In large morphs, the frontoclypeal horn is most prominent and the greatest pronotal width is larger in comparison with individuals of *C. cornifer* (Figure 5K and Figure 9A). In the lectotype, the anterior edge of pronotum is slightly emarginated at middle, forming two lobes, as well as in the specimen from Colombia. The lateral edges of the basal portion of tegmen is somewhat thickened and flattened, a condition more evident in the specimen from San Luis de Palenque (Casanare, Colombia). 

**Distribution.** *Ceracis hastifer* is the species with the least number of known localities, and there are accurate data only for three: “Tapurucuara, Rio Negro” (Manaus, Brazil), and Marabá (Pará, Brazil), in the Amazon biome; and San Luis de Palenque (Casanare, Colombia), Savannah biome, where *C. hastifer* is syntopic with *C. furcifer* (Figure 2). Labels of the lectotype and other examined historic material citing Colombia and Peru do not have further details. Aside from these, there are three specimens from São Roque (São Paulo, Brazil), of which one is a male and two are females, but due to the poor condition of male terminalia sclerites after dissection, we could not confirm identification as *C. hastifer*. 

#### 3.2.4. *Ceracis ruficornis* Pic, 1916

**Figure 10:**
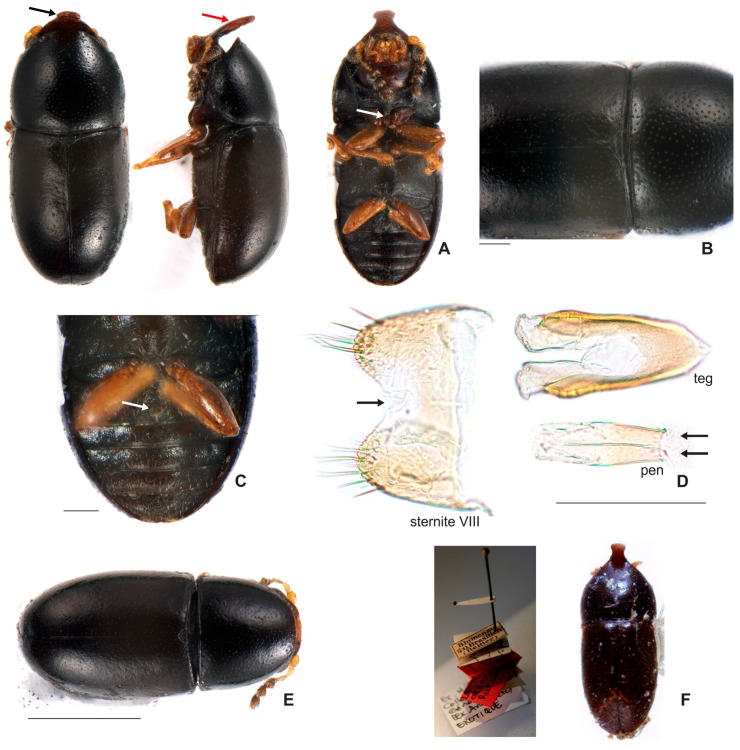
***Ceracis ruficornis* Pic. A−C** Male plesiotype, (**A**) dorsal, lateral and ventral views, respectively. Frontoclypeal ridge strongly produced forming a long horn (black arrow); laminar in lateral view (red arrow); prosternal process thin (white arrow), (**B**) pronotal punctation, (**C**) first abdominal ventrite with a sex patch at the centre (white arrow), (**D**) terminalia of a male from Bertioga, SP, Brazil, showing sternite VIII, with a deep concave emargination at the middle of the posterior edge (arrow), tegmen (teg), penis (pen) with basal edge blunt (arrows), (**E**) female plesiotype from Praia da Boraceia (SP, Brazil), (**F**) male lectotype deposited in the Muséum National d’Histoire Naturelle, MNHN (Paris, France). Scale bars: A = 0.5 mm, B−D = 0.1 mm, E,F = 0.5 mm.

Figure 5M,N; Figure 10A-F and Figure 11.4’ 

*Ceracis ruficorne* Pic, 1916: 20. Type locality: Blumenau, Brésil [13]. 

*Ceracis ruficornis* Pic, 1916: Blackwelder 1945: 550 [14] {distribution}; Lawrence 1967: 97 [3] {taxonomic status}; 1973: 202 [21] {feeding habits}; Abdullah 1973: 201 [15] {taxonomic status and distribution} Lopes-Andrade 2002: 7 [43] {taxonomic notes}; Gumier-Costa et al., 2003: 359 [9] {host fungus and distribution}; Antunes-Carvalho and Lopes-Andrade 2011: 61 [5] {taxonomic notes}; Pecci-Maddalena 2014: 482 [7] {taxonomic notes}. 

**Diagnosis.** The male frontoclypeal horn has an expanded apex, slightly emarginated at middle forming two small rounded lobes. The lateral contours of the horn do not have inflection near base but the horn is slightly narrowed at middle. The antennal funicle has three antennomeres, the first equal or longer than the following three antennomeres together. The apical portion of tegmen has an inner emargination shallowly enlarged and less than one half of the length of tegmen; the lateral edges are expanded before apex, with a shallow inflection at the outer apical edge of each lobe.

**Redescription. Plesiotype (Figure 10A−D).** Adult male apparently fully pigmented. Measurements (in mm): TL 1.22, PL 0.49, PW 0.56, EL 0.73, EW 0.59, GD 0.51. Ratios: PL/PW 0.88, EL/EW 1.24, EL/PL 1.49, GD/EW 0.86, TL/EW 2.07. **Body** glabrous, elongate, subcylindrical, shiny, blackish on dorsum; venter blackish, except for yellowish-brown legs, mouthparts, funicle and basal antennomeres; procoxae and head (including frontoclypeal horn) mostly reddish-brown; antennal club dark reddish-brown. **Head** barely visible from above; area immediately above horn base concave, glabrous, sparsely punctate; frontoclypeal ridge strongly produced forming a long horn directed upwardly (in mm: length 0.29, basal width 0.29) (Figure 10A, black arrow), laminate in lateral view (Figure 10A, red arrow), with apex expanded and slightly emarginated at middle forming two small rounded lobes (Figure 11.4’, black arrow); lateral contours of horn without inflection near base but slightly narrowed at middle. **Antennae** (right antennae measured) with FL 0.07, CL 0.16, CL/FL 2.29, length of antennomeres I−VIII (in mm) as follows: 0.06, 0.04, 0.04, 0.02, 0.01, 0.04, 0.04, 0.08; sensillifers visible. **Eyes** coarsely faceted, with minute slender yellowish setae in intersections of ommatidia; GD 0.11 mm. **Pronotum** with anterior portion produced forward, concealing head when seen from above; anterior edge slightly emarginated, forming two very short rounded lobes; sides narrow, not visible from above; posterior edge sublinear; punctation fine, single, uniform and regularly distributed (Figure 10B); distance between punctures from about 2 to 5 puncture-widths; each puncture bearing a yellowish decumbent minute seta, barely visible even at a magnification of 150x; interspaces of punctures microreticulate. **Scutellum** small, subtriangular, with few punctures, each one bearing a short, fine, decumbent seta, barely visible even at a magnification of 150x; BW 0.08 mm and SL 0.04 mm. **Elytra** about 1.5x as long as pronotum; sides subparallel at the basal two-thirds and then abruptly converging toward apex; punctation single, similar to that of pronotum, but comparatively finer; vestiture similar to that of pronotum; humeral calli conspicuous. **Metathoracic wings** developed, apparently functional. **Prosternum** in front of coxae shallowly biconcave, microreticulated; prosternal process thin (Figure 10A, white arrow), similar to *Cer. cornifer* species (Figure 4A) and parallel-sided, almost as long as coxae and projected below prosternal disc. **Hypomera** biconcave, reticulated, bearing decumbent setae; lateral contours slightly emarginated. **Pro-, meso- and metathoracic tibiae** like those of other *furcifer* group species (see diagnosis of the group). **Metaventrite** moderately convex, subglabrous, microreticulate, bearing scattered slender setae; discrimen not visible. **Abdominal ventrites** microreticulate, punctation shallow and vestiture of scattered slender setae longer than those on dorsum; length of ventrites I−V (in mm, from base to apex of each ventrite at the longitudinal midline): 0.17, 0.06, 0.07, 0.07, and 0.06. First abdominal ventrite bearing a circular, marginated pubescent sex patch at the centre (Figure 10C, arrow), similar to *Cer. cornifer* species (Figure 4B), with a transverse diameter of 0.03 mm. **Male abdominal terminalia** (Figure 10D) with posterior edge of **sternite VIII** bearing a deep, concave, mesal emargination (Figure 10D, sternite VIII, arrow); posterior corners sclerotised and rounded, bearing bristles; median portion membranous; lateral edges diverging from posterior to anterior portion; anterior edge biconcave. **Tegmen** (Figure 10D, teg and Figure 11.4’, teg) with the inner emargination of the apical portion shallowly enlarged and less than one half of the tegmen length; lateral edges expanded before apex (Figure 11.4’, teg, small red arrows), with a shallow inflection at the outer apical edges of the lobes (Figure 11.4’, teg, big red arrow). **Penis** elongate, subcylindrical; basal edge blunt (Figure 10D, pen, arrow); sides subparallel.

**Females** (Figure 10E). Like males but devoid of abdominal sex patch and horn on head. They mostly resemble females of *C. furcifer*, but are usually homogeneously black.

**Variation.** Males, measurements in mm (n = 9, including the plesiotype) TL 1.11−1.29 (1.21 ± 0.05); PL 0.41−0.51 (0.46 ± 0.03); PW 0.5−0.58 (0.54 ± 0.02); EL 0.7−0.8 (0.76 ± 0.04); EW 0.54−0.61 (0.57 ± 0.02); GD 0.46−0.53 (0.49 ± 0.02); HL 0.09−0.3 (0.21 ± 0.08). Ratios: PL/PW 0.78−0.9 (0.84 ± 0.04); EL/EW 1.24−1.45 (1.31 ± 0.06); EL/PL 1.49−1.95 (1.66 ± 0.14); GD/EW 0.82−0.91 (0.86 ± 0.02); TL/EW 2−2.2 (2.1 ± 0.6). Females, measurements in mm (n = 7) TL 1.17−1.31 (1.23 ± 0.04); PL 0.42−0.5 (0.45 ± 0.02); PW 0.5−0.57 (0.53 ± 0.02); EL 0.72−0.85 (0.78 ± 0.05); EW 0.53−0.62 (0.57 ± 0.02); GD 0.47−0.51 (0.49 ± 0.01). Ratios: PL/PW 0.79−0.94 (0.85 ± 0.05); EL/EW 1.24−1.49 (1.37 ± 0.07); EL/PL 1.44−2.02 (1.76 ± 0.16); GD/EW 0.82−0.92 (0.86 ± 0.03); TL/EW 2.09−2.23 (2.16 ± 0.6). Specimens of the following localities were measured: **Brazil:** Rio de Janeiro (Nova Friburgo and Paraty, “Ponta Negra”); São Paulo (Bertioga, “Praia da Boraceia”).

**Material examined. Lectotype male, here designated (MNHN, Figure 10F)** “[Blumenau. S.O. Brasilien. (Reitter)]\[*Ceracis* (antennes 8 art. Probabl.)]\[Type] yellow\[Type] red; [*Ceracis ruficorne* Pic]\Lectotype.\[Blumenau. S.O. Brasilien. (Reitter)]\[*Ceracis* (antennes 8 art. Probabl.)]\[Type] yellow\[Type] red; [*Ceracis ruficorne* Pic]\Paralectotype”. **BRAZIL:** 23 specimens (CELC, including one dissected male) “Brasil: SP, Praia da Boraceia, 13.ix.2011, Sandoval-Gómez, V.E. leg.\ex. *Pycnoporus sanguineus*”; 11 specimens (MZSP, including one dissected male) “Ilha dos Buzios, São Paulo, Brazil, x.1963\Exped. Dep. Zool.\SP\ *Ceracis ruficornis* Pic Det. J.F. Lawrence”; 2 specimens (MZSP, including one dissected male) “Peruibe, SP, 28.xi/01.xii.1984, Exp. MZUSP col.\*Ceracis ruficornis* Pic Gen 1”; 4 specimens (3 ANIC; 1 CELC, dissected male) “*Ceracis* (8) *ruficornis* Pic 447\Ilha da Victoria, S. Paulo, Brasil, 16-27.iii.1964\Exped. Dep. Zool.”; 4 specimens (CELC, including one dissected male) “Brasil: RJ, Nova Friburgo, xi.2003, Grossi, E.J. leg.\*Ceracis furcifer* Mellié 1848; det. C. Lopes-Andrade”; 4 specimens (CELC, including one dissected male) “Brasil: RJ, Paraty, Ponta Negra, 5-6.iii.2011, Sandoval, V.E & Idrobo, C.J. leg”.

**Remarks.** We examined photographs of the lectotype (e.g., Figure 10F). It differs from the specimens we had in our hands mainly in the anterior edge of pronotum, which is conspicuously emarginated instead of slightly emarginated as in the plesiotype. Specimens with the greatest pronotal width (PW) are the largest but smaller than the examined *C. hastifer*. Individuals of *C. ruficornis* are usually black, except for a single specimen from Paraty (Brazil-RJ), which is dark reddish-brown. As in other species of the *furcifer* group, the male frontoclypeal horn also varies in shape and length between morphs and, as in *C. cornifer*, the horn may be bifurcate at apex in small individuals with small horns (Figure 5N). However, a bifurcate male horn is also common in large morphs (Figure 5M), which is different from that observed in *C. cornifer*. The type locality of *C. ruficornis* is Blumenau (SC, Brazil), and in the examined material the closest locality was Peruíbe (SP, Brazil). However, specimens from Bertioga (SP, Brazil) were in better condition, so we used this for the redescription (one of them as a “plesiotype”). 

**Distribution.** Known only for a few localities close to the southeastern coast of Brazil, where it is sympatric with *C. cornifer* in Paraty (in the state of Rio de Janeiro), Peruíbe and Ilha da Victoria (in the state of São Paulo) (Figure 2).

#### 3.2.5. Identification Key to Males of the *furcifer* Group

1 Prosternal process laminate (Figure 11.1, white arrows); tegmen with lateral lobes narrow, apex acute (Figure 11.1, teg, black arrow).…**Other *Ceracis* species, not included here.**

1’ Prosternal process thin but not laminate (Figure 11.2, white arrows); tegmen with a deep, longitudinal, apical emargination forming two parallel lobes with rounded or blunt apices (Figure 11.2, teg, black arrow) (*furcifer* group)….**2**

2 Antennal funicle with three antennomeres (Figure 11.3)….**3**

2’ Antennal funicle with four antennomeres (Figure 11.3’)….**4**

3 Apex of frontoclypeal horn usually widened and mesally emarginate, forming two conspicuous rounded lobes (Figure 11.4, big black arrow); lateral contours of horn without inflection near base but usually conspicuously narrowed at middle (Figure 11.4, small black arrow); tegmen with lateral edges widened preapically (Figure 11.4, teg, small red arrows), with a prominent inflection at the outer apical edge of each lobe (Figure 11.4, teg, big red arrows)….***Ceracis furcifer* Mellié**

3’ Apex of frontoclypeal horn expanded but only slightly emarginated mesally, forming two small rounded lobes (Figure 11.4’, black arrow); tegmen with lateral edges slightly widened preapically (Figure 11.4’, teg, small red arrows) but with a shallow inflection at the outer apical edge of each lobe (Figure 11.4’, teg, big red arrows)….***Ceracis ruficornis* Pic**

4 Lateral contours of frontoclypeal horn usually with a pronounced inflection near base (Figure 11.5, big black arrow); anterior portion of hypomera with outer edge slightly rounded. Figure 11.5, small black arrow); apical portion of tegmen with narrow median emargination less than one half the length of tegmen (Figure 11.5, teg, red dashed line), outer edges usually with a conspicuous inflection at first basal one half (Figure 11.5, teg, red arrow), apical portion with each lobe bearing a small denticle at the inner apical edge (Figure 11.5, teg, black arrow) and a small and shallow emargination at the outer edge (Figure 11.5, teg, blue arrow)….***Ceracis cornifer* (Mellié)**

4’ Lateral contours of frontoclypeal horn straight from the basal third, usually devoid of lateral inflections (Figure 11.5’, big black arrow); anterior portion of hypomera with outer edge broadly rounded (Figure 11.5’, small black arrow); apical portion of tegmen with deep median emargination about two-thirds the length of tegmen (Figure 11.5’, teg, red dashed line), lateral edges almost straight, without or with an inconspicuous inflection at the first basal one-third (Figure 11.5’, teg, red arrow), less prominent than in *C. cornifer*, apical portion of each lobe without a denticle at the inner apical edge and without emargination at the outer edge….***Ceracis hastifer* Mellié**

## 4. Discussion

Species in the *furcifer* group are very similar externally, even in the morphology of sclerites of male abdominal terminalia. However, there are two clear subgroups: (i) species with eight antennomeres (*C. furcifer* and *C. ruficornis*), in which males usually have the frontoclypeal horn incised at apex forming two lobes and the tegmen is widened before apex, with an inflection on both outer apical edges, (ii) species with nine antennomeres (*C. cornifer* and *C. hastifer*), in which the male frontoclypeal horn is usually truncate or shallowly emarginate apically and the tegmen is almost parallel-sided, with both outer apical lobes rounded and each bearing a shallow emargination on the outer edge. Below we discuss the observed intraspecific variation in species of the *furcifer* group, their geographic distribution based on data provided here and their specialization on the host fungus *Pycnoporus sanguineus*.

### 4.1. Intraspecific Variation: Coloration and Horn Shape in Males

There is more intraspecific variation in species of the *furcifer* group than previously observed, mainly in the shape of the male frontoclypeal horn, between males of the same or of different populations (Figure 5). Another aspect is colour variation in populations of *C. cornifer* and *C. furcifer* (Figure 6 and Figure 8). These variable traits were considered diagnostic for species of the *furcifer* group recognised before our work [1,11,12,13,42,46]. Despite these differences, examination of sclerites of male abdominal terminalia, mainly the tegmen, revealed that the *furcifer* group comprises only four species, instead of eight as accepted before. Consequently, *C. cornifer* shows a disjunct geographic distribution and the greatest intraspecific variation among species of the *furcifer* group.

The use of body colour and shape of male frontoclypeal horn for delimiting ciid species has led to several synonyms (for example, see synonymy in Lawrence [3,47]). For instance, the species name *C. semipallidus* was a clear allusion to its light coloration (see Pic [46]), which is indeed a variation observed in *C. furcifer*. Therefore, the synonymization of *C. semipallidus* and *C. furcifer* proposed by Lawrence [3] is correct.

Differences in body coloration may well be a consequence of a relatively long period of time between emergence and attainment of full pigmentation, when teneral adults are abundant in ciid populations [3]. As overlap of generations is common in ciids, the consequence is that an established population may have adults with every degree of pigmentation, from recently emerged and thus light coloured (teneral) to fully pigmented adults. Although we did not test this, it would be an explanation for part of the observed body colour variation in *furcifer* group species (Figure 6 and Figure 8). 

However, we shall consider genetic and environmental factors together, which may determine body coloration. When coloration has a genetic basis, colour may also vary with environmental conditions, especially with temperature [48]. Individuals of *C. cornifer* from similar latitudes have different body coloration, being usually yellowish or reddish-brown (Figure 6C). However, individuals of the southern tip of the distribution (in areas with the lowest mean temperatures; Figure 6A−B) are usually black (Figure 6C), being conspicuously different from those in northern latitudes. Interestingly Pic [13] described *C. simplicicornis* (a new synonym of *C. cornifer* proposed here) from Buenos Ayres as “nigro-piceus”. This record represents precisely the most southern tip of the distribution and the area with the lowest mean temperature in our study (Figure 6B,D).

### 4.2. Distribution Patterns of the furcifer Group

Species of the *furcifer* group do not overlap for most of their geographic distributional ranges (Figure 2) but there are cases of syntopy and/or sympatry in a few localities (Figure 2). *Ceracis cornifer* is sympatric with *C. ruficornis* in three localities in the coast of Brazil and syntopic with *C. furcifer* in Paranhos, all these localities being in the Atlantic Forest biome, although Paranhos is very close to the Cerrado (Brazilian savanna) of Mid-West Brazil. *Ceracis cornifer* is also syntopic with *C. furcifer* in Serra do Cipó, a locality in the east limit of the Cerrado in Southeast Brazil, close to the Atlantic Forest biome. *Ceracis hastifer* and *C. furcifer* are syntopic in San Luis de Palenque (Colombian Savannah), and in Marabá (Amazon biome). Therefore, all syntopies observed by us are restricted to their distributional frontiers, which coincide with frontiers between biomes. Further studies shall evaluate whether the split of species of the *furcifer* group was related with the separation of the Atlantic and Amazon biomes. This event, which occurred in the Pliocene period, explains the currently distribution patterns of several organisms, e.g., mammals, birds, insects and plants [49,50,51,52,53,54,55] and is a plausible hypothesis to be tested for explaining the distribution patterns we show here. 

### 4.3. Specialization on the Host Fungus Pycnoporus sanguineus 

Although there are records of *furcifer* group in other fungi (see Table A2), they are mostly associated with *P*. *sanguineus* (Table A1). Aside from ciid beetles, few animals utilize basidiomes of *P. sanguineus* as food (Table A1). For instance, a colony of *Acromyrmex lundi* (Guérin, 1838) accepted basidiomes of *P. sanguineus* as food under laboratory conditions, although in the field it was observed harvesting basidiomes of *Agrocybe* [56]. 

The cases of consumption of *P. sanguineus* by humans were isolated and regard principally to South American indigenous tribes [57,58,59], which use *P. sanguineus* as medicine, e.g., for treating against aphtha or insolation, in both cases diluting basidiomes in water and drinking [58]. Fidalgo and Hirata [57] studied the names and uses given to *P. sanguineus* in three Brazilian indigenous tribes from the Xingu National Park, state of Mato Grosso. In only one of these indigenous tribe (Txucarramãe) there were reports of people eating *P. sanguineus*. Humans use basidiomes of *P. sanguineus* in the early decay stages [60,61], in which they are powdered and, when used to treat mouth infections, directly applied to the affected area. When basidiomes begin to turn white, they become highly toxic [61], due to toxic elements and heavy metals absorbed by basidiomes, e.g., cadmium [62]. Basidiomes of *P. sanguineus* also have high concentration of cinnabarins, toxic substances with antibiotic action [10,26]. Such high toxicity can function as a barrier for consumption by organisms. However, the use of *P. sanguineus* by ciid beetles suggests that they may be resistant to antibiotic and toxic compounds of this fungus [10]. Populations of *C. cornifer* can colonize basidiomes of *P. sanguineus* before they begin to sporulate [9], when levels of toxic substances are low. However, the population increases and individuals continue feeding when basidiomes become more toxic in advanced decay stages. Here, the number of records in *P. sanguineus* (mainly breeding records) demonstrates that the *furcifer* group constitute the only truly specialized animals feeding on basidiomes of *P. sanguineus*. Therefore, these beetles perform a great environmental service, removing basidiomes of *P. sanguineus*, which would persist in nature for years. 

One of the ecological bases for evolution of specialization is environmental constancy [63]. The use of a single fungus as resource might have put the *furcifer* group in an evolutionary stasis: in all areas occupied by their populations, they use the same resource, which is widely available in most open areas in the Neotropical region. On the other hand, populations of polyphagous species can undergo subsequent specialization, becoming oligophagous or even monophagous, which may act as evolutionary constraint and lead to rapid radiation. For instance, that was possibly the case for polyphagous ciid species of the *Ceracis cucullatus* group [5,6] and also of the *Cis taurus* group [19]. In both *C. cucullatus* and *Cis taurus* groups, there are a few widespread polyphagous species and several geographically restricted oligophagous ones [4,6,10,19]. The evolutionary stasis of the *furcifer* group is a hypothesis that may explain why disjunct populations of *C. cornifer* have not undergone speciation, or at least evolution has not lead to conspicuous morphological differences in individuals of disjunct populations. 

## 5. Conclusions

The *furcifer* group now encompasses four species: *C. cornifer C. hastifer*, *C. furcifer* and *C. ruficornis*. The male frontoclypeal horn and body coloration in these species have great intraspecific variation; therefore, these are not good diagnostic features. We concluded that examination of sclerites of male terminalia is the most accurate way to identify species of the *furcifer* group. We show that the *furcifer* group are widely distributed along the Neotropical region and constitute the only specialized animals that feed on basidiomes of *P. sanguineus*. 

## Figures and Tables

**Figure 11:**
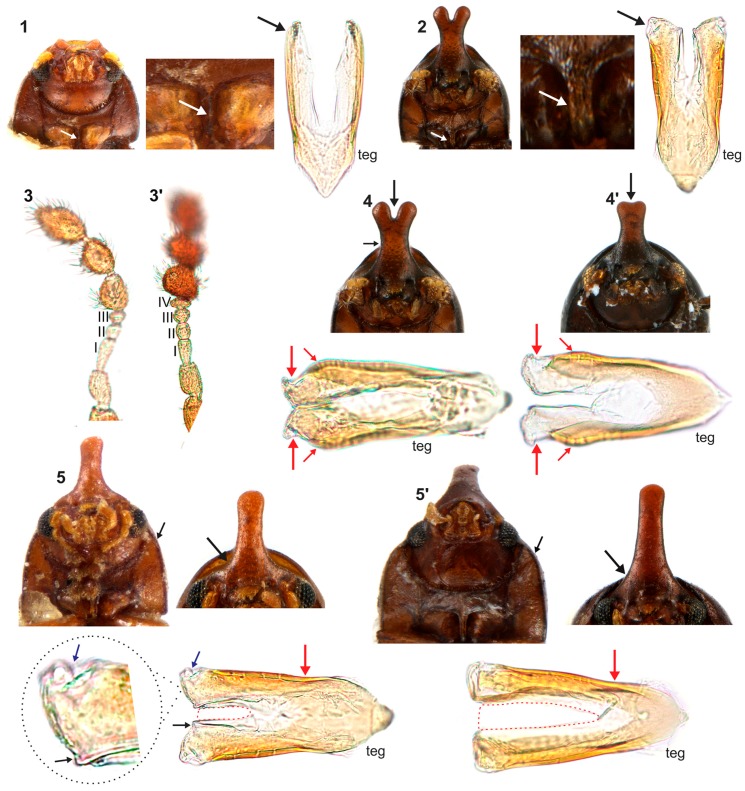
Characters for the identification of males of the *Ceracis furcifer* species-group. See 3.2.5. Identification key above for explanations.

## Appendix A

**Table A1:** List of animals feeding on basidiomes of *Pycnoporus sanguineus*. The numbering of each morphospecies cited below is the same used in the corresponding source.

Species	Record	Breeding record	Source
*Ceracis cornifer* (Mellié)	84	40	[9,10,21,64] and present work
*Cis* aff. *pusillus* (*comptus* group)	Undetermined	Undetermined	[65]
*Ceracis minutus* Dury	3 (3)	3	[21,66]
*Cis neserorum* Souza-Gonçalves & Lopes-Andrade (as *Cis* sp. E)	1	Undetermined	[67,68]
*Ceracis furcifer* Mellié	12	6	[1] and present work
*Ceracis hastifer* (Mellié)	1	0	Present work
*Ceracis ruficornis* Pic	1	1	Present work
*Homo sapiens* L.	4	NA **	[57,58,59,60]
Insects (without details)	1	Undetermined	[69]
*Acromyrmex lundi* (Guérin-Méneville) *	1	Undetermined	[56]
*Ceracis* sp.2	1	Undetermined	[64]
*Cis* sp.3 (*taurus* group)	1	Undetermined	[64]
*Strigocis vicosensis* Lopes-Andrade	1	Undetermined	[64]
*Xylographus contractus* Mellié	1	Undetermined	[64]
*Xylographus rufipes* Pic	1	Undetermined	[64]
*Ceracis tabellifer* (Mellié)	3	Undetermined	[6]
*Strigocis* sp.	3	Undetermined	[10]
*Cis* sp.4 (*tricornis* group)	1	Undetermined	[10]
*Grossicis diadematus* (Mellié)	1	Undetermined	[10]
*Cis* sp.2 (*vitulus* group)	1	Undetermined	[10]
*Cis* sp.3 (*comptus* group)	1	Undetermined	[10]
*Ceracis cucullatus* (Mellié)	1	Undetermined	[6]
*Ceracis punctulatus* Casey	2	Undetermined	[21,47]
*Cis creberrimus* Mellié	1	Undetermined	[21,47]
*Cis subfuscus* Gorham	Undetermined	Undetermined	[47]
*Strigocis opacicollis* Dury	1	Undetermined	[21,47]

* Accepted *P. sanguineus* under laboratory conditions. ** Not applicable.

**Table A2:** Other host fungi of the *furcifer* group.

Species	Host fungus	Record	Source
*Ceracis cornifer* (Mellié)	*Lenzites betulina* (L.) Fr.	1	[10]
*Ceracis cornifer* (Mellié)	*Trametes villosa* (Sw.) Kreisel	3	[64]
*Ceracis cornifer* (Mellié)	*Phellinus gilvus* (Schwein.) Pat.	1	[64]
*Ceracis cornifer* (Mellié)	*Stereum* sp.	1	[64]
*Ceracis cornifer* (Mellié)	*Schizophyllum commune* Fr.	1	[64]
*Ceracis cornifer* (Mellié)	*Trametes* sp.	1	[65]
*Ceracis cornifer* (Mellié)	*Trametes* sp.	1 (one breeding record)	Present work
*Ceracis cornifer* (Mellié)	*Trametes hispida* Bagl.	1	Present work
*Ceracis furcifer* Mellié	*Coriolus* sp. and *Lenzites* sp.	1	[21]
*Ceracis furcifer* Mellié	*Daedalea elegans* Spreng.	1	Present work
*Ceracis furcifer* Mellié	*Polyporus maximus* (Brot.) Fr.	1	Present work
*Ceracis furcifer* Mellié	*Daedalea elegans* Spreng.	1	Present work
*Ceracis ruficornis* Pic	*Coriolus* sp. and *Lenzites* sp.	1	[21]
